# ZBTB20 is required for anterior pituitary development and lactotrope specification

**DOI:** 10.1038/ncomms11121

**Published:** 2016-04-15

**Authors:** Dongmei Cao, Xianhua Ma, Jiao Cai, Jing Luan, An-Jun Liu, Rui Yang, Yi Cao, Xiaotong Zhu, Hai Zhang, Yu-Xia Chen, Yuguang Shi, Guang-Xia Shi, Dajin Zou, Xuetao Cao, Michael J. Grusby, Zhifang Xie, Weiping J. Zhang

**Affiliations:** 1 Department of Pathophysiology, Second Military Medical University, 800 Xiangyin Road, Shanghai 200433, China; 2 Department of Pathophysiology, Dalian Medical University, 9 West Section, Lvshun South Road, Dalian, 116044, China; 3 Department of Cell Biology, Second Military Medical University, 800 Xiangyin Road, Shanghai 200433, China; 4 Department of Endocrinology, Changhai Hospital, 168 Changhai Road, Shanghai 200433, China; 5 Barshop Institute for Longevity and Aging Studies, University of Texas Health Science Center at San Antonio, 15355 Lambda Drive, San Antonio, Texas 78245, USA; 6 National Key Laboratory of Molecular Biology and Department of Immunology, Chinese Academy of Medical Sciences, 9 Dongdan Santiao, Beijing, 100005, China; 7 Department of Immunology and Infectious Diseases, Harvard School of Public Health, 651 Huntington Avenue, Boston, Massachusetts, 02115, USA

## Abstract

The anterior pituitary harbours five distinct hormone-producing cell types, and their cellular differentiation is a highly regulated and coordinated process. Here we show that ZBTB20 is essential for anterior pituitary development and lactotrope specification in mice. In anterior pituitary, ZBTB20 is highly expressed by all the mature endocrine cell types, and to some less extent by somatolactotropes, the precursors of prolactin (PRL)-producing lactotropes. Disruption of Zbtb20 leads to anterior pituitary hypoplasia, hypopituitary dwarfism and a complete loss of mature lactotropes. In ZBTB20-null mice, although lactotrope lineage commitment is normally initiated, somatolactotropes exhibit profound defects in lineage specification and expansion. Furthermore, endogenous ZBTB20 protein binds to *Prl* promoter, and its knockdown decreases PRL expression and secretion in a lactotrope cell line MMQ. In addition, ZBTB20 overexpression enhances the transcriptional activity of *Prl* promoter *in vitro*. In conclusion, our findings point to ZBTB20 as a critical regulator of anterior pituitary development and lactotrope specification.

## Abstract (web summary)

The pituitary is a complex structure with the anterior lobe containing five specialized cell types secreting different hormones. Here Cao *et al*. unravel a role for ZBTB20 in pituitary development and specifically in lactrotrope specification.

## Graphical abstract

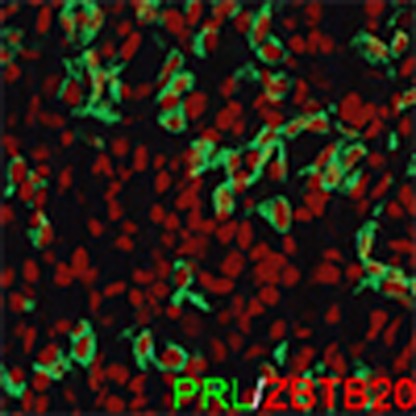

The pituitary gland functions as a relay between hypothalamus to peripheral target organs and plays a central role in regulating homeostasis, metabolism, growth, reproduction and lactation^1^,^2^. It is composed of two anatomically and functionally different entities, the adenohypophysis, including the anterior and intermediate lobes, and neurohypophysis, also known as the posterior lobe. The anterior lobe harbours five distinct specialized cell types defined by trophic hormones they generate and secrete. Somatotropes produce growth hormone (GH); lactotropes secrete prolactin (PRL); thyrotropes generate thyroid stimulating hormone (TSH); gonadotropes produce luteinizing hormone (LH) and follicle-stimulating hormone (FSH); and corticotropes secrete adrenocorticotropin hormone (ACTH).

Pituitary organogenesis is a complexly regulated and highly coordinated process both in time and space. While the neurohypophysis is derived from the neural ectoderm, the adenohypophysis originates from Rathke's pouch, which is an invagination of the oral ectodermin response to neural epithelium signals^2^. The proliferative progenitors in definitive Rathke's pouch undergo gradual differentiation and migration, and eventaully give rise to the five endocrine cell types in the anterior pituitary at birth. During early postnatal life, the anterior lobe expands rapidly, mainly caused by the stimulation of cell proliferation by trophic hormones from hypothalamus. In the developing pituitary, many transcription factors are temporally and spatially regulated, and critically involved in the lineage specification and differentiation^2^.For example, Pitx1, Pitx2, Lhx3, Prop1 and Pax6 control the early phases of pituitary patterning, while Pit-1 (Pou1f1), SF1, Gata2 and Tbx19 regulate cell differentiation^1^,^3^. It has been reported embryonic stem cells can be stimulated by these factors to differentiate into various endocrine cells *in vitro*^4^, while mutations in these transcription factors have been linked with congenital hypopituitarism and several combined or isolated pituitary hormone deficiencies. Particularly, Pit-1 is required for the differentiation of three lineages: thyrotropes, somatotropes and lactotropes. Pit-1 expression begins at embryonic day 13.5 (E13.5) in mice and maintains in thyrotropes, somatotropes and lactotropes throughout adulthood. In a cell-specific manner, Pit-1 regulates the transcription of genes encoding hormone products, such as TSH, GH, PRL and growth hormone-releasing hormone receptor (GHRHR)^1^,^5^,^6^,^7^. Therefore, mutations with the *Pit1* gene are associated with combined GH, PRL and TSH deficiency^8^,^9^,^10^.

The ontogeny of lactotropes is closely related with somatotrope lineage, and highly regulated by hormones and growth factors. Lactotropes are scarce in the fetal anterior pituitary, and begin to increase at postnatal day 3 (P3) in mice, following the appearance of GH-expressing cells. Notably, the early lactotropes also express GH in the rat and cow^11^. Importantly, a majority of lactotropes are depleted in the transgenic mice using rat GH promoter to target the destruction of GH-expressing cells^12^,^13^, thus establishing a notion that a majorty of lactotropes arise from the dual hormone-secreting somatolactotropes. However, how lactotropes are specified from somatolactotropes is still an enigma. There are some evidence that oestrogen, insulin-like growth factor 1 (IGF1) and fibroblast growth factor 2 (FGF2) regulate the cell proliferation and differentiation of lactotroptes, but none of these factors are essential for lactotrope specification. Treatment of fetal pituitary with 17β-estradiol *in vitro* increases the number of PRL-positive cells^14^. Disruption of either oestrogen receptor alpha gene (*Era)* or *Igf1* leads to a marked reduction both in PRL expression and lactotrope cell number, however, with no obvious effects on lactotrope specification^15^,^16^,^17^. Although FGF2 is capable of initiating differentiation of lactotropes rather than somatotropes from a Pit1-expressing progenitors *in vitro*^18^, no obvious pituitary phenotypes have been reported in *Fgf2* knockout mice^19^,^20^,^21^. Therefore, the regulatory mechanism about lactotrope development remains unclear.

ZBTB20 belongs to a subfamily of zinc finger proteins containing C2H2 Krüppel-type zinc fingers and BTB/POZ domains^22^. It plays important roles in multiple systems, as suggested by the severe phenotypes of the mice lacking ZBTB20, which mainly include growth retardation, premature lethality, infertility and hypoglycemia^23^,^24^,^25^,^26^. Noteworthy, many of the null phenotypes implicate a possibility of hypopituitarism, however, little is known about its role in pituitary biology. In this study, we show that ZBTB20 is highly expressed by all the mature endocrine cell types in anterior pituitary, and its deficiency leads to anterior pituitary hypoplasia and hypopituitarism as a result of complete absence of mature lactotropes and impaired expansion of somatotropes. ZBTB20-null mice display sever defects in lactotrope specification and expansion despite the normal initiation of its lineage commitment, whereas the mice lacking Zbtb20 specifically in nervous system show normal lactotrope development. ZBTB20 knockdown in MMQ lactotrope cells results in a robust decrease in PRL expression and secretion. Furthermore, we demonstrate that ZBTB20 binds to *Prl* promoter and enhances its transcriptional activity. Thus our findings for the first time establish a critical role of ZBTB20 in anterior pituitary development and lactotropic lineage specification.

## Results

### Hypopituitarism in ZBTB20-deficient mice

**Figure 1:**
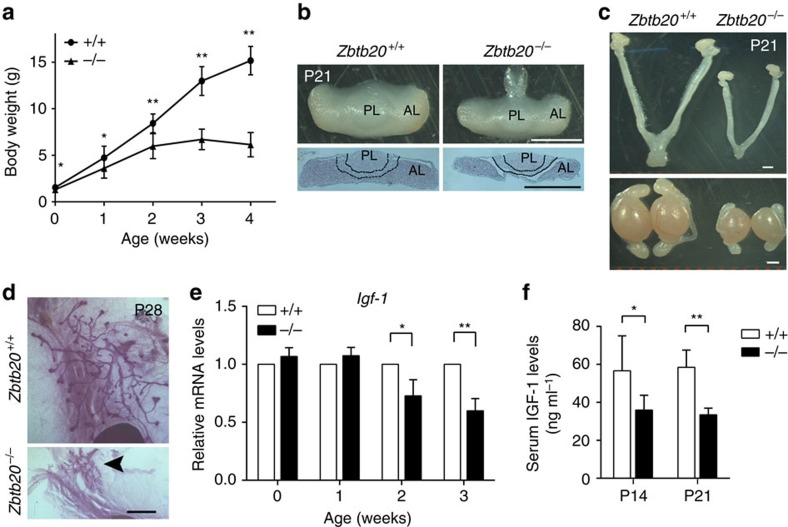
Hypopituitarism in global ZBTB20-null mice. (**a**) Body weight of *Zbtb20*^+/+^ and *Zbtb20*^−/−^ mice. *n*=11–20 per time point. Values represent mean±s.e.m. *P<0.05 versus +/+, **P<0.01 versus +/+ (Student's *t*-test.) (**b**) A general view (upper) and section (lower, H&E) of 21-day-old pituitaries from *Zbtb20*^+/+^ and *Zbtb20*^−/−^ littermate. *Zbtb20*^−/−^ mouse has normal sized posterior lobe (black outline), but a severely reduced anterior lobe.PL, posterior lobe of pituitary; AL, anterior lobe. Scale bar, 1 mm. (**c**) Uterus and ovaries from female mice (upper) and testes and epididymides from male mice (lower) are significantly reduced in 21-day-old *Zbtb20*^−/−^ mice compared with wild-type controls. Scale bar, 1 mm. (**d**) Whole-mammary-gland mounts staining of 28-day-old *Zbtb20*^+/+^ and *Zbtb20*^−/−^ littermate. The *Zbtb20*^−/−^ mice displayed an impeded ductal elongation (black arrowhead). Scale bar, 1 mm. (**e**) Quantitative RT–PCR analysis of *Igf-1* mRNA level in liver from *Zbtb20*^+/+^ and *Zbtb20*^−/−^ mice. Results are presented as fold induction relative to mRNA level in wild-type mice. *n*=3 per time point. Values represent mean±s.e.m. *P<0.05, **P<0.01 (Student's *t*-test). (**f**) Circulating IGF-1 levels are detected by ELISA at P14 and P21. *n*=6 per time point. Values represent mean±s.e.m. *P<0.05, **P<0.01 (Student's *t*-test).

To evaluate the potential role of ZBTB20 in pituitary biology, we first examined pituitary development and function in ZBTB20-null mice. As previously described^24^, *Zbtb20* global knockout mice were grossly indistinguishable from their control littermates at birth, but exhibited significant postnatal growth retardation, premature mortality and infertility, which are indicative of hypopituitarism. At the mixed genetic background of C57BL/6J and 129 Sv, more than half of homozygous null mutants can survive up to 4 weeks, with a few to adulthood^24^. However, after backcrossed to C57BL/6J mice for 12 generations, a vast majority of mutant homozygotes died within 4 weeks of age. Therefore, we phenotypically analysed the mutant mice mainly within 3 weeks of age, when they were relatively healthy, however, with a dramatic reduction in body size and weight compared with littermate controls (Fig. 1a). At birth, there was no gross difference of pituitary between mutant and control littermates (Supplementary Fig. 1). From 1 week after birth, the mutant pituitaries were markedly smaller in size than littermate controls, thus they were only ∼50% of the normal weight at postnatal day 21 (P21; Fig. 1b). Of note, the hypoplasia was mainly observed in the anterior pituitaries from both male and female mutant mice, while their posterior lobes were not significantly affected. In addition, the female mutants also displayed profound hypoplasia in ovary and uterus (Fig. 1c). Whole mount of mammary glands revealed a severely impeded ductal elongation in 4-week-old null female mice, while age-matched control mice displayed extensive ductal elongation and branching (Fig. 1d). The hypothalamus that control pituitary development and function was anatomically and histologically normal in mutant mice. Taken together, the above observed growth retardation, sexual infantilism and anterior pituitary hypoplasia strongly suggest that ZBTB20 disruption results in hypopituitary dwarfism.

To assess GH deficiency in the mutants, we measured liver and serum levels of IGF-1, which are GH-dependent, given that random circulating levels of GH do not accurately reflect GH sufficiency due to the pulsatile nature of its secretion^27^. Compared with control mice, the mutants showed an ∼50% decrease in both liver messenger RNA (mRNA) and serum protein levels of IGF-1 at P21 (Fig. 1e,f). Combined with the anterior pituitary hypoplasia, these data indicate that ZBTB20-null mice at least have GH deficiency.

### Impaired anterior pituitary development in ZBTB20-null mice

**Figure 2:**
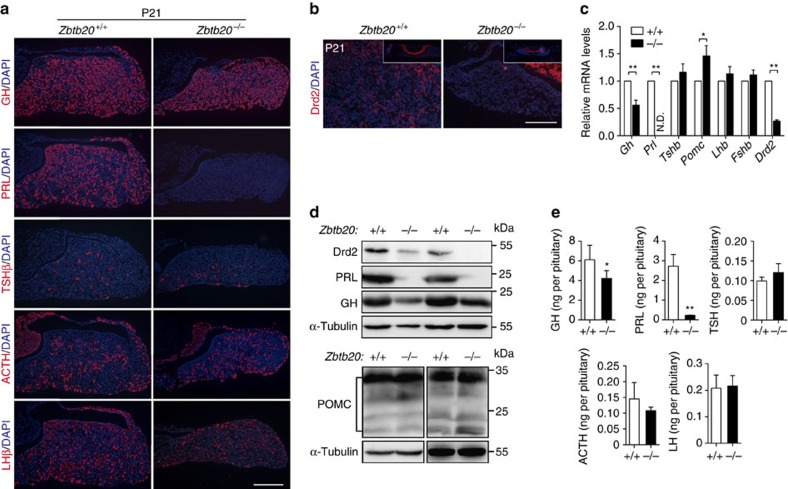
Impaired development of endocrine cells in the anterior pituitary of *Zbtb20*-null mice. (**a**) All five types of cell lineages in anterior pituitary are detected by immunohistochemical staining using GH, PRL, TSHβ, ACTH and LHβ antibodies in 21-day-old *Zbtb20*^+/+^ and *Zbtb20*^−/−^ littermate. PRL-positive cells are absent in null mice, but other cell lineages have no apparent changes. (**b**) Immunohistochemical staining of Drd2 in 21-day-old pituitary sections also shows a loss of positive cells in the null mice. Insets show the whole coronal section of pituitaries. Scale bar, (**a**) 200 μm and (**b**) 100 μm. (**c**) Quantification of hormone mRNA levels in 14-day-old *Zbtb20*^+/+^ and *Zbtb20*^−/−^ mice by real-time RT–PCR. Results are presented as fold induction relative to mRNA levels in wild-type mice. A sample was pooled from three to five adenohypophyses of the same phenotype, and the experiment was repeated three times. Values represent mean±s.e.m. *P<0.05, **P<0.01 (Student's *t*-test). (**d**) Western blot of hormones and Drd2 proteins in pituitary extracts from 14-day-old *Zbtb20*^+/+^ and *Zbtb20*^−/−^ mice. A sample was pooled from five to eight adenohypophyses of the same phenotype, and the experiment was repeated two times. (**e**) Anterior pituitary hormonal contents are measured in pituitary extracts by ELISA at P21. *n*=3. Values represent mean±s.e.m. *P<0.05 versus +/+, **P<0.01 versus +/+ (Student's *t*-test).

To determine the specific deficits in the mutant pituitary, we examined the alteration of its cell composition. Hematoxylin and eosin (H&E) staining revealed a marked decrease in the number of granule-filled acidophilic cells (somatotropes and lactotropes) in the mutant pituitary compared with control at P21 (Supplementary Fig. 2), while basophilic cells (thyrotrophs, adrenocorticotrophs and gonadotrophs) were morphologically normal. We then performed immunohistochemical staining using antibodies specifically against individual hormone produced by each cell type. At P21, there were no apparent differences in the cell densities of GH-, TSH-, LH- or ACTH-immunoreactive cells between mutant and control mice (Fig. 2a). Very strikingly, PRL-immunoreactive cells were completely absent in mutant pituitary at the same age, in contrast to their abundant distribution in normal anterior lobe. The absence of mature lactotropes in mutant pituitary was further confirmed by immunostaining with the antibodies against dopamine receptor D2 (Drd2; Fig. 2b), which is another differentiation marker of lactotropes^28^,^29^. Consistently, quantitative reverse transcription PCR (RT–PCR) and western blotting analysis revealed that PRL expression was undetectable at either mRNA or protein levels in mutant pituitary at P14 (Fig. 2c,d). In addition, GH expression was decreased by ∼50% in mutant pituitary at both mRNA and protein levels compared to control, *POMC* mRNA levels increased by 1.46-fold, while *LH* and *FSH* mRNA levels were similar between both groups at P14 (Fig. 2c). Furthermore, measurement of the hormone contents in whole pituitary extracts by ELISA revealed that GH contents were decreased by 30% in mutants, PRL contents almost undetected, and TSH, ACTH and LH contents were not altered at P21 (Fig. 2e). These data suggest that ZBTB20 disruption selectively leads to a severe developmental defect of lactotropes, and to some less extent, somatotropes are affected as well.

### ZBTB20 is essential for lactotrope lineage specification

**Figure 3:**
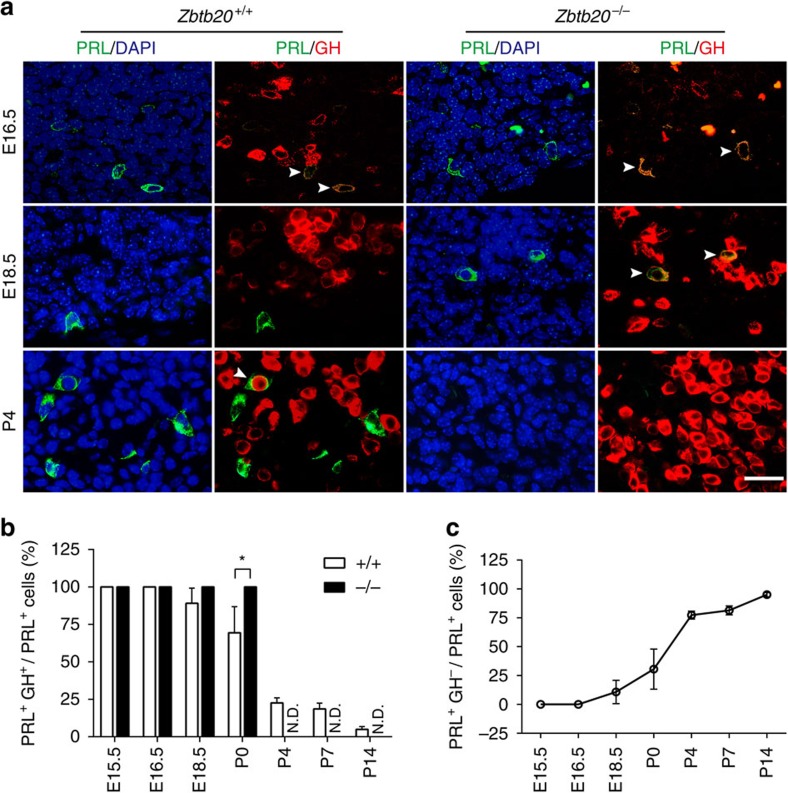
Defect of lactotropes specification and differentiation in the absence of ZBTB20. (**a**) Immunohistochemical double staining of PRL (green) and GH (red) in the pituitaries from *Zbtb20*^+/+^ and *Zbtb20*^−/−^ mice at the age from E16.5 to P4. Cell nuclei were stained with DAPI (blue). Arrowheads indicate PRL and GH double-positive cells. Scale bar, 25 μm. (**b**,**c**) The percentages of double-positive cells (**b**) and PRL single-positive cells (**c**) among the PRL-positive cells in the pituitaries of wild-type mice are shown by diagram. E15.5: *n*=28 sections from four animals; E16.5: *n*=23 sections from five animals; E18.5: *n*=15 sections from four animals; P0: *n*=21 sections from five animals; P4: *n*=26 sections from five5 animals; P7: *n*=24 sections from four animals; and P14: *n*=13 sections from two animals. Values represent mean±s.e.m. *P<0.05 versus control (Student's *t*-test).

To further characterize the developmental defect of lactotrope lineage in ZBTB20-null mice, we examined its ontogeny during embryonic and early postnatal periods. The lactotrope lineage emerges at E15.5 in mice, characterized by the expression of PRL in a restricted medial zone adjacent to the ventral surface of the intermediate lobe^2^, and undergoes rapid expansion during P7 to P21. Thus we first detected PRL expression in the developing pituitary. Consistently, immunohistochemical staining revealed that PRL-reactive cells were not detected at all in the mutant pituitary from P4 to P7 (Fig. 3a; Supplementary Fig. 3a). In addition, *Prl* mRNA expression was hardly detectable by RT–PCR after P2 (Supplementary Fig. 3b). Surprisingly, sparse PRL-immunoreactive cells were transiently detected in mutant pituitaries from E15.5 to birth, with no significant difference compared to control group (Fig. 3a; Supplementary Fig. 4). Moreover, *Prl* mRNA expression was also detected in mutant pituitary at birth, although the levels were decreased by ∼70% compared with control (Supplementary Fig. 3b). These data suggest that lactotrope lineage could be transiently initiated in Zbtb20-null pituitary in the perinatal period.

Given the crucial role of dual hormone-expressing somatolactotrope progenitors in lactotrope development^13^, we then analysed the generation and differentiation of somatolactotropes by double immunostaining of GH and PRL. At E15.5 and E16.5, just like control mice, nearly all of PRL-expressing cells were GH-positive in mutant pituitaries, implying the apparently normal generation of somatolactotropes in the absence of ZBTB20 (Fig. 3a; Supplementary Fig. 4). Notably, in control pituitary, PRL single-positive cells (PRL^+^GH^−^), which represent the specified lactotropes, began to emerge at E18.5, and gradually increased in the number and percentage to PRL-positive cells as the pituitary developed from P0 to P14. As a result, PRL^+^GH^-^ lactotropes account for over 75% of PRL-positive cells at P4 and P7, while double-positive cells (GH^+^PRL^+^) <25% (Fig. 3b,c). At P14, the vast majority of PRL-positive cells (>95%) were PRL^+^GH^−^, and only a few (<5%) were double-positive cells. In contrast, the mutant PRL-positive cells were consistently double-positive somatolactotropes from E15.5 to P0 (Fig. 3a,b; Supplementary Fig. 4), and completely vanished after P3, while PRL^+^GH^−^ cells were not detected in the mutant pituitary during the early development. These data suggest that ZBTB20 may be not required for the initial commitment of somatolactotropes, but is indispensable for their specification into lactotropes.

### Impaired cell proliferation in ZBTB20-null anterior pituitary

**Figure 4:**
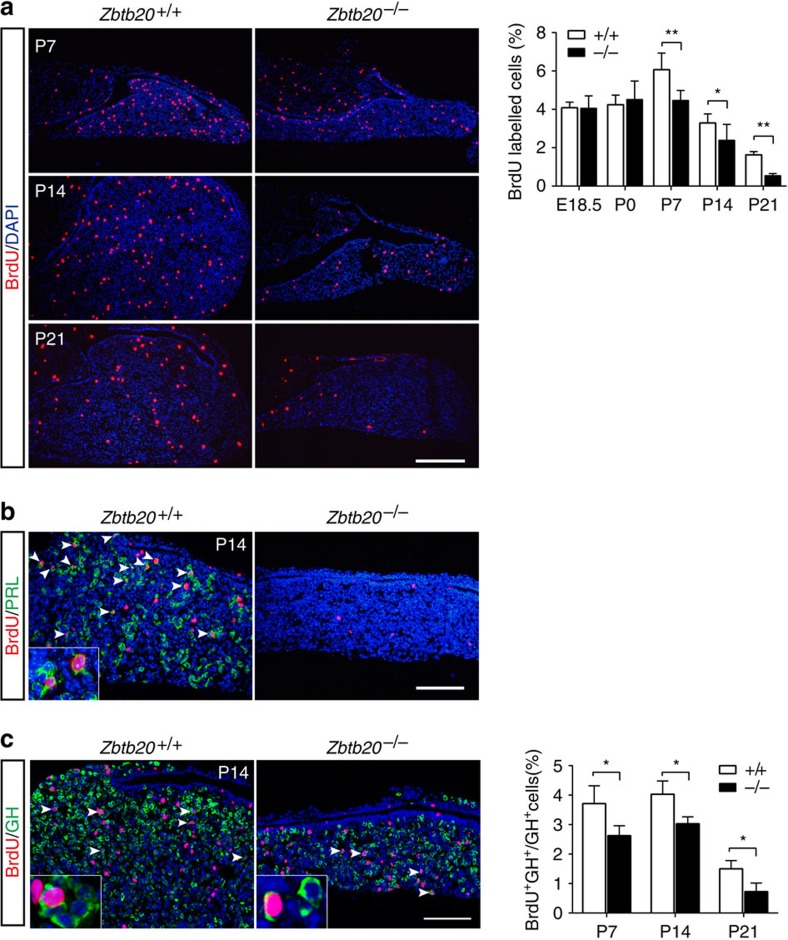
Impaired postnatal cell proliferation in *Zbtb20*^−/−^ pituitary. (**a**) Immunohistochemical staining of BrdU was performed on E18.5 to P21 pituitaries of *Zbtb20*^+/+^ and *Zbtb20*^−/−^ littermates to assess cell proliferation. The percentage of BrdU-positive cells in anterior lobe was determined from counting serial sections of each pituitary. E18.5: *n*=20 sections from four animals; P0: *n*=26 sections from seven animals; P7: *n*=31 sections from five animals; P14: *n*=34 sections from five animals; and P21: *n*=27 sections from four animals. Values represent mean±s.e.m. *P<0.05, **P<0.01 (Student's *t*-test). (**b**,**c**) Immunohistochemical double staining of BrdU (red) and PRL (**b**) or GH (**c**) (green) was performed on the pituitary at the indicated age (P7 to P21). Arrowheads indicate double-positive cells, and inserts show the enlargement of double-positive cells. The percentage of double-positive cells among the GH-positive cells was determined by counting serial sections of each pituitary. P7: *n*=8 sections from three animals; P14: *n*=12 sections from three animals; and P21: *n*=9 sections from three animals. Values represent mean±s.e.m. *P<0.05 (Student's *t*-test). Scale bar, (**a**) 200 μm; (**b**,**c**) 100 μm.

To investigate the cellular basis of pituitary hypoplasia in ZBTB20-null mice, we detected cell apoptosis and proliferation in postnatal pituitary. TUNEL staining revealed that very rare TUNEL-positive apoptotic cells were detected in the developing pituitaries from P7 to P21, with no significant difference between control and mutant mice (Supplementary Fig. 5). Then the proliferation rate was evaluated by measuring bromodeoxyuridine (BrdU) incorporation after a single intraperitoneal injection. There was no significant difference of BrdU incorporation into anterior pituitary cells between the two genotypes at E18.5 or P0 (Supplementary Fig. 6a). However, in comparison with control, the density of BrdU-positive cells was reduced in the mutant anterior pituitary by ∼27% (*n*=31–34 slices from five animals) at P7 and P14, and more dramatically, by about 67% (*n*=27 slices from four animals, *P*<0.01, Student's *t*-test) at P21 (Fig. 4a), indicating a defect of cell proliferation in the mutant pituitary.

To further characterize the cell proliferation of lactotropes and somatotropes in the developing pituitary, we did double immunostaining with anti-BrdU and anti-GH or anti-PRL antibodies, respectively. During P7 to P21, when the mutant pituitary were devoid of PRL-positive cells, double staining of BrdU and PRL revealed that the control mice exhibited efficient BrdU incorporation in PRL-positive cells, indicative of robust lactotrope lineage expansion (Fig. 4b). In addition, Zbtb20 deficiency also resulted in profound decrease in BrdU incorporation of somatotropes in the same period (Fig. 4c; Supplementary Fig. 6b). These data strongly indicate that pituitary hypoplasia in ZBTB20-null mice is primarily caused by the loss of lactotrope lineage development and the impairment of somatotrope expansion as well.

During postnatal development, the expansion of anterior pituitary originates from a proliferation of both already differentiated endocrine cells and stem/progenitor cells in the stimulation of proliferative signals. To determine whether the impaired cell proliferation was due to the defect of postnatal pituitary progenitors, we detected Sox2 expression, which is a marker of pituitary progenitors^30^,^31^. Immunohistochemical analysis revealed that the number and distribution of Sox2-positive cells were not significantly affected in anterior pituitary at P21 by the loss of ZBTB20 (Supplementary Fig. 7). These data suggest that the impaired cell proliferation in ZBTB20-null pituitary is not caused by the deficit of postnatal anterior pituitary progenitors, rather than the defect of differentiated endocrine cells.

### Defects of lactotropic lineage expansion in ZBTB20-null mice

**Figure 5:**
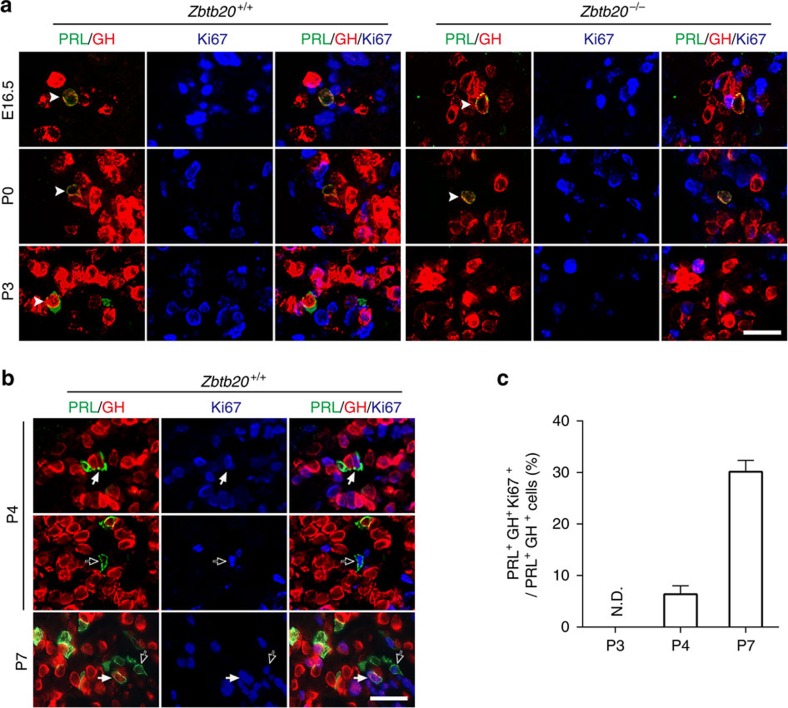
Characterization of the proliferation of somatolactotropes from control and Zbtb20-null mice. (**a**,**b**) Triple immunostaining of GH (red), PRL (green) and Ki67 (blue) was performed on the pituitaries from control and mutant mice at the indicated age. Arrowheads (**a**) indicate PRL^+^GH^+^ double-positive cells, which are Ki67 negative. Solid white arrows (**b**) indicate PRL^+^GH^+^ double-positive cells expressing Ki67, and open arrows (**b**) indicate PRL^+^GH^-^ cells expressing Ki67. Scale bar, 25 μm. (**c**) The percentage of Ki67-expressing PRL^+^GH^+^ cells in PRL^+^GH^+^ cells was determined by counting serial sections of each pituitary at the indicated age. P3: *n*=22 sections from five animals; P4: *n*=19 sections from four animals; and P7: *n*=18 sections from four animals. Values represent mean±s.e.m. ND, not detectable.

To evaluate the defect of lactotrope lineage expansion in the absence of ZBTB20 in more detail, we characterized somatolactotrope proliferation by triple immunostaining with the antibodies against GH, PRL and the cell proliferation marker Ki67. Unexpectedly, Ki67 expression was not detected in GH^+^/PRL^+^ cells from control mice until P4 (Fig. 5a,b), indicating a non-replicating state before P3. The Ki67-positive replicating somatolactotropes accounted for 6.5% (10/152) and 32.6% (30/92) of total GH^+^/PRL^+^ cells at P4 and P7 (Fig. 5c), respectively. Interestingly, Ki67 protein was also present in PRL^+^GH^−^ cells in control pituitary at P7 and P14 (Fig. 5b). These data suggest that both somatolactotropes and differentiated lactotropes are normally proliferative, and may contribute to the lactotrope lineage expansion in pituitary development. In contrast, ZBTB20-null somatolactotropes were consistently Ki67-negative, which transiently emerged before P3, and afterwards completely vanished before the entry into the time window of cell replication. Collectively, these data suggest that ZBTB20 is essential for the lineage specification and expansion of lactotropes.

### Characterization of Pit-1 expression in ZBTB20-null pituitary

**Figure 6:**
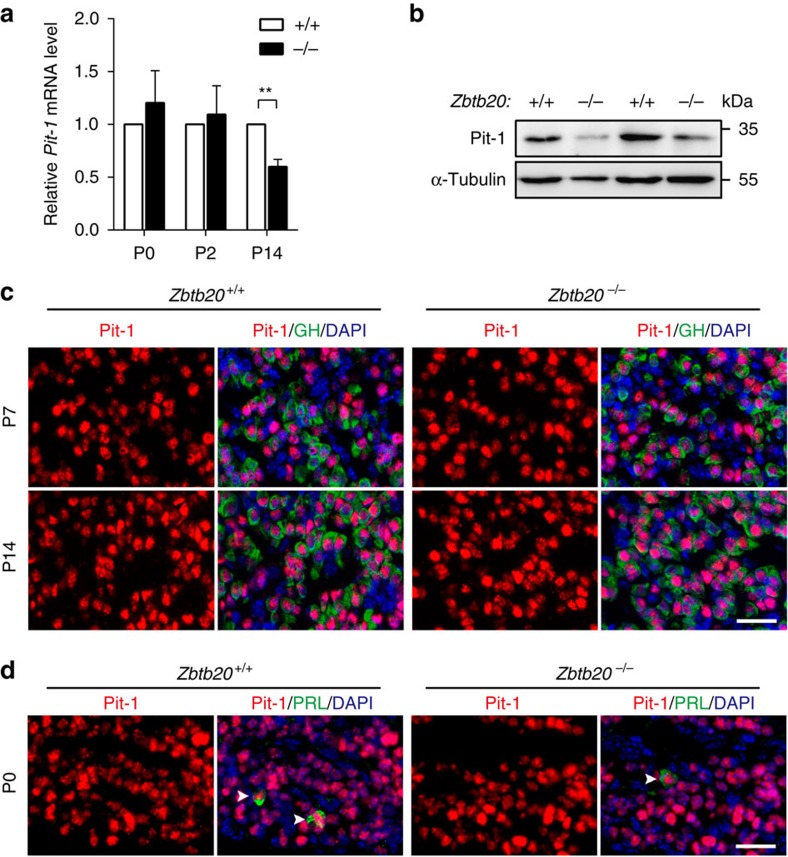
Analysis of Pit-1 expression in anterior pituitary. (**a**) Quantification of *Pit1* mRNA level at the indicated stages in *Zbtb20*^+/+^ and *Zbtb20*^−/−^ mice by real-time RT–PCR. Results are presented as fold induction relative to mRNA levels in wild-type mice. A sample was pooled from four adenohypophyses of the same phenotype, and the experiment was repeated two times. Values represent mean±s.e.m. **P<0.01 (Student's *t*-test). (**b**) Western blot of Pit1 proteins in pituitary extracts from 14-day-old *Zbtb20*^+/+^ and *Zbtb20*^−/−^ mice. A sample was pooled from five to eight adenohypophyses of the same phenotype, and the experiment was repeated twice. (**c**,**d**) Immunohistochemical double staining of Pit1 (red) and GH or PRL (green) is performed at the indicated stages in *Zbtb20*^+/+^ and *Zbtb20*^−/−^ pituitaries. Cell nuclei are stained with DAPI (blue). Arrowheads indicate PRL-positive cells. Scale bar, 25 μm.

Considering the pivotal role of Pit-1 in the lineage development of somatotropes and lactotropes, we then analysed Pit-1 expression in the mutant pituitary. RT–PCR and *in situ* hybridization analyses revealed that *Pit-1* mRNA expression levels were comparable between control and mutant pituitary at P0 and P2 (Fig. 6a; Supplementary Fig. 8a), and immunohistochemical staining showed no significant difference of Pit-1 protein expression between the two groups at E18.5 (Supplementary Fig. 8b).These data suggest that Pit-1 expression is not altered in the perinatal pituitary by the loss of ZBTB20. However, RT–PCR and western blot analyses revealed that ZBTB20 deletion led to a marked reduction in *Pit-1* expression at both mRNA and protein levels in the pituitary at P14 (Fig. 6a,b). To determine whether ZBTB20 disruption resulted in a decrease in Pit-1 expression in somatotropes *per se*, we further performed double immunostaining of Pit-1 and GH. There was no significant difference of Pit-1 staining intensity in somatotropes between the two genotypes at P7 or P14 (Fig. 6c), which suggests that decreased Pit-1 expression in mutant pituitary most likely reflects the loss of Pit-1-expressing lactotropes and somatotropes. On the other hand, Pit-1 and PRL double staining showed that Pit-1 expression in PRL-positive cells was comparable between control and mutant pituitary at birth (Fig. 6d), suggesting that the defect of lactotrope specification in the absence of ZBTB20 was not likely due to the reduction of Pit-1 expression *per se*.

### ZBTB20 expression in pituitary and hypothalamus

**Figure 7:**
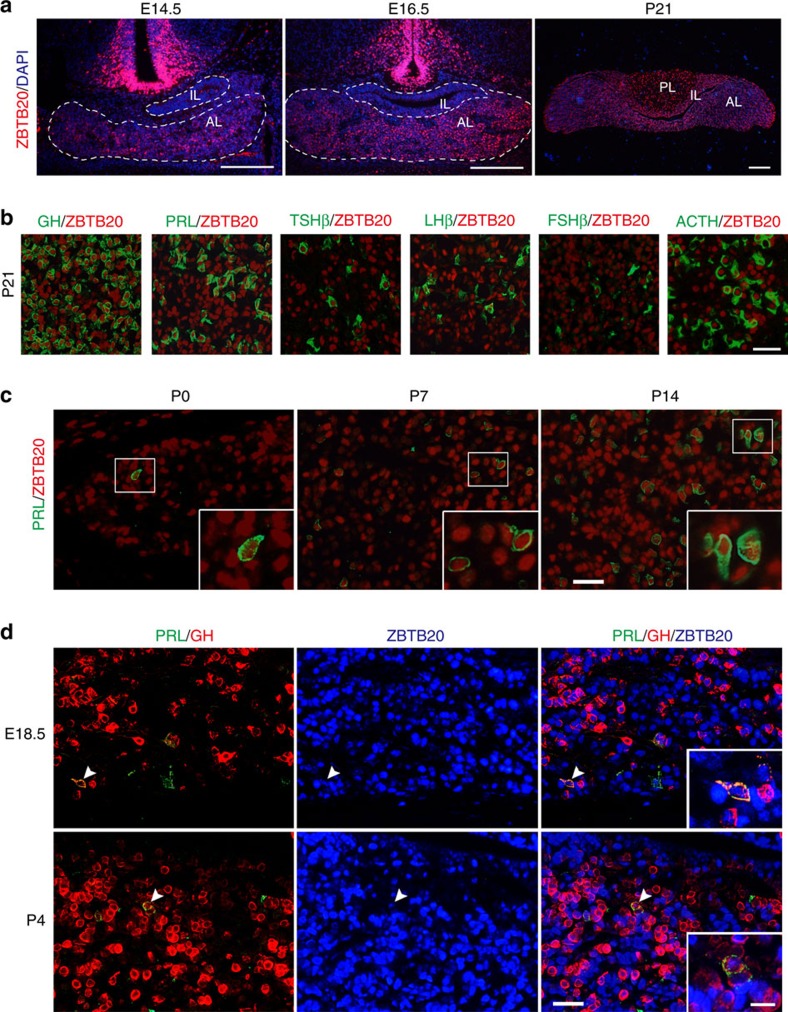
Expression of ZBTB20 in the developing hypothalamus and pituitary. (**a**) ZBTB20 expression was detected by immunohistochemical staining at the indicated age on mouse adenohypophysis. White outline indicates adenohypophysis in embryo. AL, anterior lobe; IL, intermediate lobe; PL, posterior lobe. Scale bar, 200 μm. (**b**,**c**) Immunohistochemical double staining of ZBTB20 (red) and hormones (green) in pituitary at the indicated age. Inserts are enlargement of the framed areas. Scale bar, 30 μm (outside the inserts); 10 μm (inside the inserts). (**d**) Triple staining of ZBTB20 (blue), PRL (green) and GH (red) in the pituitary at the indicated age. Arrowheads indicate PRL and GH double-positive cells expressing ZBTB20. Inserts show higher magnification of the area indicated by the arrowhead. Scale bar, 30 μm (outside the inserts); 10 μm (inside the inserts).

To understand the mechanisms by which ZBTB20 regulates anterior pituitary development and lactotrope specification, we examined its expression pattern in the developing pituitary and hypothalamus. The expression of ZBTB20 protein was first detected in hypothalamus as early as E13.5, but was excluded in pituitary at this time point (Supplementary Fig. 9). At E14.5, ZBTB20 protein was first detected in some cells of anterior pituitary, but absent in intermediate lobe (Fig. 7a). The number of ZBTB20-positive cells and intensity of ZBTB20 expression rapidly increased in the following 2 days. At E16.5, ZBTB20-positive cells were abundantly distributed over the entire anterior lobe, the expression levels of which were variable and much lower than hypothalamus. At P21, ZBTB20 expression was maintained at high levels in the pituitary (Fig. 7a). Double immunostaining of ZBTB20 and the pituitary hormones revealed that ZBTB20 was expressed by all the five endocrine cell types in anterior pituitary at P21 (Fig. 7b). Of note, most PRL-positive cells consistently expressed high levels of ZBTB20 from P0 to P14 (Fig. 7c). Furthermore, triple immunostaining with anti-GH, anti-PRL, and anti-ZBTB20 antibodies revealed high expression of ZBTB20 in somatolactotropes at both E18.5 and P4 (Fig. 7d). These data suggest that ZBTB20 might regulate anterior pituitary development and lactotrope specification in cell-autonomous and/or nonautonomous manners.

### Neuronal ZBTB20 is dispensable for lactotrope specification

Considering the role of hypothalamic dopamine in the negative regulation of lactotrope proliferation via its receptor Drd2, we first analysed the expression of the genes critically involved in dopamine synthesis and transportation. The mRNA expression levels of *Th* and *Dat*, which encode tyrosine hydroxylase and dopamine transporter, respectively, were comparable between control and mutant hypothalamus at P14 (Supplementary Fig. 10), implying that ZBTB20 deficiency might not alter the release of dopamine from hypothalamus to pituitary. To further determine whether hypothalamic ZBTB20 is involved in lactotrope specification, we inactivated this gene specifically in nervous system using Cre/loxP recombination system. The Nestin-Cre mouse line can mediate efficient gene deletion by E11 (ref. 32), a time point much earlier than the onset of ZBTB20 protein in nervous system including hypothalamus. Therefore we generated the pan-neuron-specific knockout mice of ZBTB20 (NS-ZB20KO) by crossing *Zbtb20* loxP mice onto Nestin-Cre mice^33^. Considering that this Cre line alone has some hypopituitarism in adulthood^34^, we analysed pituitary development of NS-ZB20KO mice only at early postnatal stage. Immunostaining analysis revealed that ZBTB20 expression was not affected in NS-ZB20KO pituitary, while totally abolished in the hypothalamus (Supplementary Fig. 11a). At P21, NS-ZB20KO pituitaries were smaller in size than control mice bearing *Cre* gene (Supplementary Fig. 11b). This raised the possibility that hypothalamic ZBTB20 could have a role in regulating pituitary development. However, there were no significant differences of either hormone mRNA levels or hormone-positive cell distribution between control and NS-ZB20KO mice (Supplementary Fig. 11c,d). These data at least indicate that neuronal ZBTB20 is dispensable for lactotrope specification. Thus, we reason that impaired lactotrope development in the absence of ZBTB20 is most likely due to an intrinsic defect of anterior pituitary.

### Regulation of PRL expression by ZBTB20 in lactotropes

**Figure 8:**
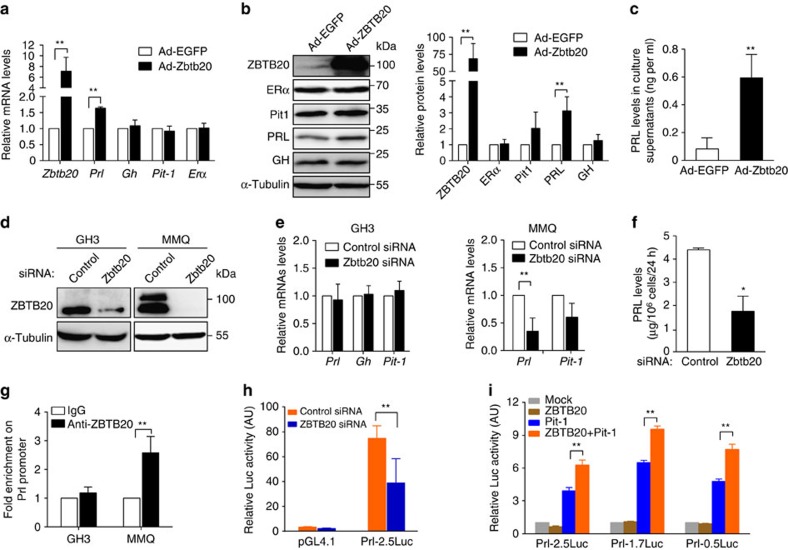
ZBTB20 regulates *Prl* expression in a cell-autonomous fashion. (**a**–**c**) ZBTB20 overexpression in GH3 cells increases PRL expression and secretion without significant effect on GH expression. GH3 cells were infected with Ad-ZBTB20 or Ad-EGFP at MOI of 400. Three days later, GH3 cells were subjected to gene expression analyses at mRNA levels by RT–PCR (**a**) and at protein levels by western blotting (**b**), with the expression levels presented as fold change relative to Ad-EGFP control, while PRL secretion was measured in the culture supernatants by ELISA (**c**). *n*=4. Values represent mean±s.e.m. **P<0.01 versus Ad-EGFP (Student's *t*-test). (**d**–**f**) ZBTB20 knockdown decreases PRL expression and secretion in MMQ but not GH3 cells. Control or ZBTB20 siRNA was introduced into GH3 or MMQ cells by electroporation, respectively. Three days after transfection, the cells were harvested for western blotting (**d**) and quantitative RT–PCR analyses (**e**), and the culture supernatants were measured for PRL levels by ELISA (**f**). *n*=4. Values represent mean±s.e.m. *P<0.05 versus control siRNA (Student's *t*-test). (**g**) ChIP assay showed the binding of ZBTB20 to rat *Prl* promoter in MMQ rather than GH3 cells. *n*=3. Values represent mean±s.e.m. **P<0.01 versus control IgG (Student's *t*-test). (**h**) ZBTB20 knockdown decreases the transcriptional activity of *Prl* promoter in MMQ cells. MMQ cells were co-transfected with siRNA and expression plasmids by electroporation. Luciferase activity was measured at 48-h post transfection, and normalized with internal control. *n*=3. Values represent mean±s.e.m. ** *P*<0.01 versus control siRNA (Student's *t*-test). (**i**) ZBTB20 overexpression enhances the transcriptional activity of the *Prl* promoters in 293T cells in the presence of Pit-1. Rat *Prl* promoters are 0.5, 1.7 kb, or 2.5 kb in length. *n*=3. Values represent mean±s.e.m. ** *P*<0.01 versus Pit-1 group (Student's *t*-test).

To explore the mechanisms by which ZBTB20 promotes lactotrope differentiation in a cell-autonomous fashion, we took advantage of a rat somatolactotrope cell line GH3 expressing both GH and PRL, and a rat lactotrope cell line MMQ, which expresses PRL but not GH. At both mRNA and protein levels, MMQ cells expressed much higher levels of *Prl* and *Zbtb20* compared with GH3 cells (Supplementary Fig. 12). Considering that it is necessary for lactotrope differentiation to maintain PRL expression meanwhile extinguishing GH expression, we first examined the potential effect of ZBTB20 overexpression on *Prl* expression and lactotropic differentiation of GH3 cells. After 72 h of infection with recombinant replication-deficient adenoviruses co-expressing ZBTB20 and EGFP (hereafter Ad-ZBTB20) at the MOI of 400, more than 85% of GH3 cells were green fluorescent protein-positive by flow cytometry assay (Supplementary Fig. 13). Compared with EGFP mock control, ZBTB20 overexpression in GH3 cells led to a marked increase in *Prl* expression and protein secretion into culture supernatants, but had no effect on GH expression at either mRNA or protein levels (Fig. 8a–c), suggesting that ZBTB20 upregulation alone is not sufficient to drive lactotropic differentiation from somatolactotropes. In addition, the expression of *Pit1* or *ER*α was not affected by ZBTB20 overexpression in GH3 cells.

We next evaluated the effect of ZBTB20 knockdown on PRL expression of these two cell lines. Small interfering RNA (siRNA) against *Zbtb20* was introduced into GH3 or MMQ cells by electroporation, which caused robust downregulation of ZBTB20 protein levels at day 3 post-treatment compared with control siRNA (Fig. 8d). Expectedly, ZBTB20 knockdown did not significantly affect the mRNA expression levels of PRL, GH or Pit-1 in GH3 cells (Fig. 8e), which is in agreement with the normal emergence of somatolactotropes in ZBTB20-null pituitary. In contrast, MMQ cells displayed a substantial decrease in *Prl* mRNA levels as well as its protein secretion in the culture supernatants as a result of ZBTB20 knockdown (Fig. 8e,f), whereas *Pit-1* expression was not significantly affected, and *GH* mRNA still undetectable. In addition, the cell growth rate of MMQ was not significantly altered by ZBTB20 knockdown. Consistently, ChIP analyses revealed the binding of endogenous ZBTB20 protein to *Prl* promoter in MMQ but not GH3 cells (Fig. 8g). Therefore, these data suggested that ZBTB20 is specifically required to maintain *Prl* gene expression in lactotropes rather than somatolactotropes, but dispensable for GH gene repression in lactotropes.

We further performed *Prl* reporter assay to determine whether ZBTB20 regulates the transcriptional activity of *Prl* promoter. ZBTB20 knockdown in MMQ cells led to a robust decrease in the rat *Prl* promoter activity (Fig. 8h). On the other hand, the reporters harbouring different length of rat Prl promoters were not active in human embryonic kidney cell line 293T due to the absence of Pit-1 protein. Overexpression of Pit-1 alone could robustly activate the *Prl* reporters. More interestingly, ZBTB20 overexpression resulted in a marked increase in the transcriptional activity of the *Prl* promoters in 293T cells in the presence of Pit-1 (Fig. 8i). These data strongly indicate that ZBTB20 regulates *Prl* gene transcription in a cell-autonomous manner.

Put together, we postulate a possible model for the regulation of lactotrope development by ZBTB20 (Supplementary Fig. 14). In this model, somatolactotropes are derived from Pit-1 lineage independently of ZBTB20, and undergo cell differentiation and proliferation before being specified into lactotropes in a ZBTB20-dependent manner. Alternatively, lactotropes can be specified from somatolactotropes before the latter undergo cell replication, or even directly derived from GH-negative Pit-1 lineage, for either of which ZBTB20 is essential. Therefore, disruption of ZBTB20 leads to complete blockage of lactotrope specification and lineage expansion.

## Discussion

In the present study, we for the first time establish that ZBTB20 is a lineage-specific regulator for anterior pituitary development. Global disruption of ZBTB20 leads to a decrease in somatotrope population and a total loss of mature lactotropes, with the manifestations of anterior pituitary hypoplasia, hypopituitary dwarfism and impaired mammary gland development, while other lineages (for example, thyrotropes, corticotropes and gonadotropes) are not significantly affected. Of note, both lactotropes and somatotropes are generated from Pit-1 lineage, however, their development is differentially regulated by ZBTB20. In the absence of ZBTB20, lactotrope development is completely blocked at the early stage of PRL^+^GH^+^ somatolactotropic precursors as a result of the defect of cell specification, whereas somatotrope development is mildly impaired mainly because of the decrease in cell proliferation. Therefore, ZBTB20 plays critical roles in both lactotrope specification and somatotrope expansion in the developing anterior pituitary.

This study further excludes the possibility that hypothalamic ZBTB20 regulates lactotrope specification. The hypothalamus begins to express ZBTB20 earlier and at much higher levels than pituitary during embryonic development, raising the possibility that hypothalamic ZBTB20 may regulate pituitary development in an extrinsic manner. However, Nestin-Cre-mediated neuron-specific ablation of ZBTB20 did not affect pituitary development and lactotrope specification during early postnatal stage. Because this Cre transgenic line itself has mild hypopituitarism in adulthood^34^, it is not an ideal model to investigate the role of hypothalamic ZBTB20 in postnatal pituitary development. Currently, using this line, we at least conclude that hypothalamic ZBTB20 is dispensable for lactotrope specification. This conclusion is also supported by the observation that the expression levels of *Th* and *Dat* were not altered in hypothalamus by the loss of Zbtb20, which are involved in dopamine synthesis and transportation. On the other hand, ZBTB20 is expressed by somatolactotropes in the developing anterior pituitary. Therefore, most likely, it is pituitary ZBTB20 that regulates lactotrope specification. To more precisely determine the role of hypothalamic ZBTB20 in pituitary development, other neuron-specific Cre lines, e.g. directed by endogenous Tau promoter^35^, may be a good option. On the other hand, knockdown of ZBTB20 in lactotrope cell line MMQ results in a robust decrease in PRL protein expression and secretion. Furthermore, ZBTB20 could bind to and regulate the transcriptional activity of *Prl* promoter in MMQ cells. These data strongly suggest that ZBTB20 most likely regulates lactotrope specification in a cell-autonomous manner.

The ontogeny of lactotropes is closely related with somatotropes. The early lactotropes also express GH in the rat and cow^11^. Importantly, a majority of lactotropes are depleted in the transgenic mice using rat GH promoter to target diphtheric toxin A-mediated destruction of GH-expressing cells^12^. Similarly, both somatotropes and lactotropes are largely depleted in the GH promoter-driven thymidine kinase transgenic mice after consecutive administration of the synthetic nucleoside FIAU (1-(2-deoxy-2-fluoro-β-δ-arabinofranosyl)-5-iodouracil) to kill dividing cells during embroynic and postnatal development^13^. These observations strongly suggest that a majorty of lactotropes may arise from the dual hormone-expressing somatolactotropes. However, the molecular mechanism about lactotrope specification has not been defined. We found that ZBTB20 expression begins to emerge at E14.5 in pituitary. Of note, ZBTB20 is also expressed by somatolactotropes, and ZBTB20 deficiency leads to complete blockage of lactotrope specification at the stage of somatolactotropes. In ZBTB20-null mice, PRL^+^GH^+^ precursors are consistently present between E15.5 to P0, but display a complete defect to differentiate into PRL^+^GH^−^ cells, which occurs as early as at E18.5 in control mice. These data suggest that ZBTB20 is essential for lactotrope specification from GH-expressing precursors. Alternatively, lactotropes can also develop independently of somatotrope lineage^12^,^36^. However, as a result of ZBTB20 disruption, PRL single-positive cells were not detected in either embryonic or postnatal pituitary. Therefore, we reason that ZBTB20-regulated cell differentiation is critical for lactotrope specification in both somatolactotrope-dependent and -independent pathways.

Our study further points out that ZBTB20-regulated cell differentiation is critical for lactotrope lineage expansion. The previous transgenic study show that proliferating GH-positive cells are critical for postnatal lactotrope development^13^; however, the dynamics of somatolactotrope proliferation has not been characterized so far. Our data demonstrate that differentiating somatolactotropes are not replicating until P4. From P7 to P14, somatolactotropes undergo robust cell proliferation, which to great extent accounts for the lactotrope lineage expansion. Therefore, lactotrope specification can occur before or after somatolactotrope replication. In addition, specified lactotropes are also highly proliferating at P14, which may also contribute to lactotrope lineage expansion. ZBTB20 deletion leads to a complete blockage of lactotrope lineage expansion due to the failure of somatolactotropes to enter the cell replication window and lineage specification. The fate of ZBTB20-null somatolactotropes could be apoptosis. However, due to the scarcity of the cell population in the perinatal pituitary, it is hard to trace somatolactotropic apoptosis. Alternatively, they could be differentiated to other lineages than lactotropes, for example somatotropes. To confirm this possibility, lineage tracing experiment needs to be performed in the future.

Pit-1 is crucial for the development of somatotropes, lactotropes and thyrotropes. Pit-1 mutation causes the combined deficiency of the three lineages. Particularly, in response to oestrogen, ERα promotes PRL expression by synergistic interaction with Pit-1, and largely accounts for the postnatal expansion of lactotropes^7^,^37^,^38^. However, ablation of ERα has no effects on lactotrope specification despite of a decrease in PRL expression levels and lactotrope cell number^16^. We found that *Pit-1* expression was not significantly altered in ZBTB20-null lactotropic progenitors compared to control at P0, raising a possibility that ZBTB20 may regulate lactotrope specification through interaction with Pit-1. Our reporter assay revealed that ZBTB20 could enhance the transcriptional activity of *Prl* promoter in the presence of Pit-1, suggesting that ZBTB20 may be critically involved in the activation of *Prl* gene by Pit-1 probably as a coactivator. In the previous studies, we have established ZBTB20 as a transcriptional repressor of the genes encoding alpha-fetoprotein (AFP) in liver^23^,^39^, fructose-1,6,-bisphosphotase 1 (Fbp1) in pancreatic β cells^26^, IκBα in macrophages^40^, and Sox9 in hypertrophic chondrocytes^41^. Put together, we thus postulate that the transcriptional regulatory functions of ZBTB20 could be context-dependent. Finally, it is also possible that ZBTB20 regulates the expression of other un-identified targets than *Prl* gene, which are critical for lactotrope development. Therefore, the exact molecular mechanism by which ZBTB20 regulates pituitary development and lactotrope specification needs further investigation in the future.

In summary, ZBTB20 is required for anterior pituitary development and lactotrope specification. Our identification of ZBTB20 as a crucial determinant of lactotrope specification provides insight into the ontogeny of lactotropes, which will help to unravel the cellular and molecular basis for pituitary deficiency.

## Methods

### Animals

ZBTB20-null mice were generated by crossing between *Zbtb20*^+/−^ mice^24^, and central nervous system-specific *Zbtb20* knockout mice were generated by repeated crossing *Zbtb20*^*flox*^ mice with Nestin-cre transgenic mice^23^,^33^. Mice were bred in specific pathogen-free conditions. The mice used for the experiments were homozygous mutant mice and their wild-type littermates, and backcrossed into the C57BL/6 background for a minimum of 10 generations. Because a vast majority of homozygous *Zbtb20*^−/−^ mice die within one month of age, experiments were performed in mice within 3 weeks of age. All experiments were performed in accordance with the guidelines of the Second Military Medical University Animal Ethics Committee.

### Tissue preparation

The morning when vaginal plug was observed was designated as embryonic day 0.5 (E0.5), and the day of birth was P0. For morphological analysis, embryos were collected from timed-pregnant females, and their heads were fixed with 4% paraformaldehyde overnight and embedded in paraffin. Mouse pups were anaesthetized by hypothermia (P0–P4) or 5% urethane (30 μl per g of body weight; P7–P28), and perfused transcardially with 4% paraformaldehyde. Pituitary glands were dissected out by stereo microscope, immersed in 4% paraformaldehyde for 40 min to 2 h, and embedded in paraffin. Consecutive serial sections (4 μm) at 100-μm intervals were collected for immunostaining.

### Cell proliferation and apoptosis assays

For BrdU experiments, timed-pregnant mice and postnatal 0, 7, 14 and 21 days old mice were injected intraperitoneally with BrdU (100 mg per kg of body weight) and killed 2 h later for immunostaining analysis using anti-BrdU antibody. For apoptosis assays, serial coronal pituitary sections were subjected to TUNEL staining following the manufacturer's protocol (Promega).

### Histology and immunohistochemistry

H&E staining of pituitary and whole-mount analysis of mammary gland were performed according to standard protocols^42^. Pituitary or hypothalamus sections were deparaffinized, rehydrated, blocked in PBS with bovine serum albumin and incubated with primary antibody. The following primary antibodies were obtained from Dr A.F.Parlow at the National Institutes of Health of USA to identify pituitary cell lineages: rabbit anti-GH, -PRL, -TSHβ, -FSHβ and -ACTH antibodies. For single immunofluorescent staining, tissue sections were incubated overnight at 4 °C with the above hormone antibodies (1:2,000), anti-LHβ antibody (1:2,000, Acris), anti-ZBTB20 antibody 9A10 (home-made, 1:2,000), anti-Drd2 antibody (1:1,000, Millipore), anti-Sox2 antibody (1:2,000, Santa Cruz), anti-BrdU antibody (1:2,000, Sigma-Aldrich), and anti-Pit1 antibody (1:500, Abcam). The signals were amplified by TSA Plus DNP system (thereafter TSA system, NEN Life Science Products Inc., Boston)^25^, and visualized with Alexa Fluor 594. For hormones and ZBTB20 or BrdU double labelling, pituitary sections were incubated with different hormone antibodies (1:1,000, NHPP) and anti-ZBTB20 antibody 9A10 (1:1,000) or anti-BrdU antibody (1:2,000), signals were amplified by TSA system, and visualized with Alexa Fluor 594 and 488, respectively. For PRL and GH colabelling, pituitary sections were incubated with goat anti-PRL antibody (1:100, Santa Cruz) and rabbit anti-GH antibody (1:100, NHPP), followed by incubation with Alexa Fluor 594- or Alexa Fluor 488-conjugated secondary antibodies (1:200, Molecular Probes, Life Technologies), respectively. For Pit-1 and PRL or GH double labelling, pituitary sections were incubated with mouse anti-Pit1 antibody (1:250, Abcam) and rabbit anti-PRL or –GH antibodies (1:100, NHPP). Pit-1 signal was amplified by TSA system and visualized with Alexa Fluor 594, while PRL and GH signals were visualized by incubation with Alexa Fluor 488-conjugated secondary antibodies. For triple staining of PRL/GH/Ki67 or PRL/GH/ZBTB20, pituitary sections were incubated with goat anti-PRL antibody (1:100, Santa Cruz), rabbit anti-GH antibody (1:100, NHPP) and rat anti-Ki67 antibody (1:1,000, Dako) or mouse anti-ZBTB20 antibody 9A10 (1:1,000). Ki67 and ZBTB20 signals were amplified by TSA system and visualized with Alexa Fluor 350, while PRL and GH signals were visualized by incubation with Alexa Fluor 488- and Alexa Fluor 594-conjugated secondary antibodies, respectively.

### *In situ* hybridization

After perfused with 4% paraformaldehyde in PBS, pituitary glands of mice were fixed with 4% paraformaldehyde for 2 h and saturated with 20% sucrose in PBS overnight at 4 °C. Pituitary were embedded in optimal cutting temperature (OCT) compound and sectioned at 10 μm. Antisense riboprobe of *Pit-1* labelled with digoxygenin-UTP were transcribed from complementary DNA (cDNA) clone. After overnight hybridization with riboprobe, coronal pituitary sections were detected with anti-digoxygenin (Roche) antibody conjugated to alkaline phosphatase, and developed by nitro blue tetrazolium.

### Quantitative RT–PCR

Adenohypophyses were isolated under stereo microscope and pooled per genotype. Total RNA was extracted from adenohypophyses, livers, and hypothalamus using TRIzol reagent (Invitrogen, Life Technologies), and treated with DNase I (Fermentas, Thermo). cDNAs were produced with a ReverTra Ace-a Kit (Toyobo), and real-time PCR was performed with a QuantiFast SYBR Green PCR Kit (Qiagen). Primer sequences are listed in Supplementary Table 1. Data were normalized to *Gapdh* expression and expressed as the fold increase of control.

### Western blotting

Adenohypophyses pooled per genotype were lysed in RIPA buffer supplemented with protease inhibitor mixture. Protein concentrations of the extracts were measured with BCA protein assay kit (Picerce, Thermo). Proteins were resolved on 4–12% gradient SDS/PAGE, transferred to PVDF membranes, and immunoblotted with anti-ZBTB20 clone 9A10 antibody (1:2,000), anti-PRL antibody (1:1,000, NHPP), anti-GH antibody (1:1,000, NHPP), anti-POMC antibody (1:500, NHPP), anti-Pit1 antibody (1:500, Abcam), anti-DRD2 antibody (1:500, Millipore), and anti-α-Tubulin antibody (1:5,000, Proteintech). Secondary antibodies (1:10,000, Vector) coupled to horseradish peroxidase were used, and blots were developed with a West Pico enhanced chemiluminescence kit (Pierce, Thermo). Uncropped scans are shown in Supplementary Fig. 15.

### ELISA for IGF-1 and pituitary hormones

For IGF-1 measurement, blood was collected by retro-orbital puncture under 5% urethane anaesthesia after 6-h fasting. Plasma IGF-1 levels were measured by using a mouse IGF-1 ELISA kit (Alpco, Salem, NH). For pituitary hormones measurement, a single adenohypophysis was lysed in 100 μl RIPA buffer and diluted by PBS to 1 ml. Anterior pituitary hormones were measured with corresponding mouse ELISA kits (Alpco).

### Cell counts

To quantify proliferating cells, BrdU-positive cells were counted randomly in a minimum of 600 anterior pituitary cells from the coronal sections. Three to seven sections were selected from each pituitary; and four to seven animals were used for each time point. The total number of positive cells was expressed as the percentage with the same area. To quantify PRL^+^GH^−^ cells, PRL^+^GH^+^cells and proliferating PRL^+^GH^+^cells, all PRL-positive cells, PRL^+^GH^+^ cells and triple positive cells on coronal sections were counted. Two to six sections were selected from each animal, and two to five animals were used for each time point.

### Cell culture and adenoviral infection

Rat GH3 and MMQ cells were obtained from American Type Culture Collection (ATCC), and cultured in F-12K Nutrient Mixture (Gibco, Life Technologies) supplemented with 15% horse serum, 2.5% fetal bovine serum, and 1% penicillin/streptomycin. Recombinant adenoviral vector expressing ZBTB20 was constructed by inserting human ZBTB20 cDNA into pAd-track (Stratagene) under the control of CMV promoter, and *in vitro* recombination with adenoviral backbone vector pAdEasy-1 and subsequent viral packaging in 293A cells were performed according to the instructions of the manufacturer. Recombinant adenoviruses were purified by CsCl ultracentrifugation, and subjected to dialysis against PBS before titration in 293A cells. GH3 cells were infected with recombinant adenoviruses co-expressing ZBTB20 and EGFP (Ad-ZBTB20) or mock adenoviruses expressing EGFP (Ad-EGFP) at MOI of 400, and incubated for 3 days for the expression of ZBTB20 before harvesting for analysis. The efficiency of infection was assessed by measurement of percentage of EGFP-positive cells using a flow cytometry (Becton Dickinson).

### RNA interference analysis

After wash with PBS twice, 5 × 10^5^ GH3 or MMQ cells were resuspended in BTXpress High Performance Electroporation solution (Harvard Apparatus, MA, USA) containing 2.5 μmol of control or Zbtb20 siRNA (Dhmarcon, PA, USA), and subjected to electroporation in a 2-mm cuvette using a BTX ECM830 electroporator^41^, with the setting of one pulse of 170 volts for 15 ms. Then cells were plated in normal culture media. Twenty-four hours after transfection, the cells were collected by centrifugation, and resuspended in fresh media with the density of 1 × 10^5^ ml^−1^. After culture for another 48 h at 37 °C, the culture supernatants were collected for PRL measure with ELISA kit (Alpco), and the cells were subjected to mRNA and protein expression analyses.

### Chromatin immunoprecipitation analysis

Fragmented chromatin from GH3 or MMQ cells was incubated overnight with anti-ZBTB20 antibody 9A10 (home-made, 0.5 μg per 10^6^ cells), anti-acetylated histone 3 (Millipore, 0.5 μg per 10^6^ cells) as positive control, or isotype IgG (Upstate, 0.5 μg per 10^6^ cells) as negative control, and followed by incubation with Dynabead-conjugated Protein G (Invitrogen). Purified chromatin DNA was subjected to real-time PCR analysis with the primers for rat *Prl* promoter, the sequence of which is 5′-TTTGGGGTCAGAAGAGGC-3′ and 5′-TTGTGGAAGGAGCGCAGT-3′. The data were analysed using the formula of 2^–ΔΔCt^, where ΔΔCT=(*C*t[IP]–*C*t[input])_SA_– (*C*t[IP]–*C*t[input])_NS_. SA=Specific antibody, NS=Non-specific antibody. Three independent ChIPs were performed.

### Promoter luciferase reporter assay

A *Prl* promoter-driven firefly luciferase reporter (#608) was first constructed by cloning rat *Prl* promoter region (covering from −2486 to +65, named PRL-2.5Luc) into the pGL4.10 vector (Promega) at the sites of *SacI* and *Bgl2*, the sequence of which was confirmed by DNA sequencing. Truncated Prl reporters were obtained by the removal of 5′ end of Prl promoter from the reporter PRL-2.5Luc with restrictive digestion, thereby containing ∼1.7 or 0.5 kb of *Prl* proximal promoters (Prl-1.7Luc and Prl-0.5Luc), respectively. MMQ cells were co-transfected with control or ZBTB20 siRNA (2.5 μmol) plus 500 ng of reporter plasmids by electroporation. HEK 293 T cells (ATCC) were co-transfected in 24-well plate with reporter constructs, ZBTB20 and/or Pit-1 expression plasmids using Effectene transfection reagent (Qiagen). The pGL4.1 empty vector served as a blank control. Renilla luciferase plasmid RL-TK was co-transfected as internal control to normalize the luciferase activity. Forty-eight hours after transfection, the cells were collected, and disrupted with passive lysis buffer (Promega). Luciferase activity was measure using a Dual-Luciferase Reporter Assay System (Promega) on a GloMAX luminometer (Promega), and firefly luciferase activity was normalized to renila luciferase activity of internal control. Experiments were performed in triplicate and repeated three times.

### Statistical analysis

Quantitative data are shown as mean±s.e.m. Differences among more than two groups were performed by two-way analysis of variance, and differences between two groups were performed with Student *t*-test. *P* values of <0.05 were considered statistically significant.

## Additional information

**How to cite this article:** Cao, D. *et al*. ZBTB20 is required for anterior pituitary development and lactotrope specification. *Nat. Commun.* 7:11121 doi: 10.1038/ncomms11121 (2016).

## Supplementary Material

Supplementary Information Supplementary Figures 1-15 and Supplementary Table 1
