# Supplementary material for: ZBTB20 is required for anterior pituitary development and lactotrope specification
Source: Nat Commun. 2016 Apr 15;7:11121. doi: 10.1038/ncomms11121 (PMC4835541; doi:10.1038/ncomms11121)
Supplement: Supplementary Information — Supplementary Figures 1-15 and Supplementary Table 1 [file ncomms11121-s1.pdf]

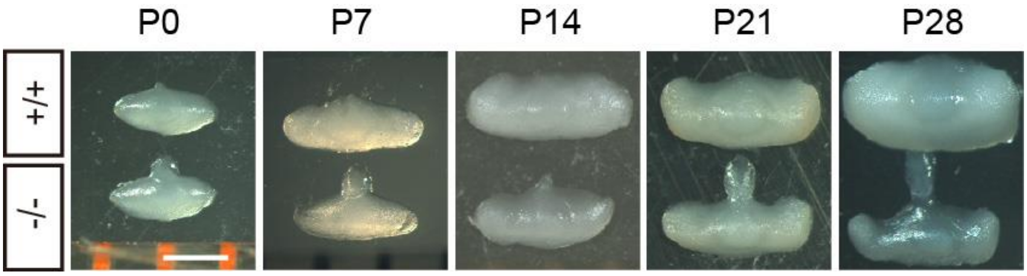

Supplementary Figure 1. Pituitary hypoplasia in *Zbtb20*$^{-/-}$ mice. Gross morphology of pituitaries at different stages shows the decreased volume of pituitaries in null mice occurs from one week after birth compared with those of wild-type littermates. Scale bar: 1 mm.

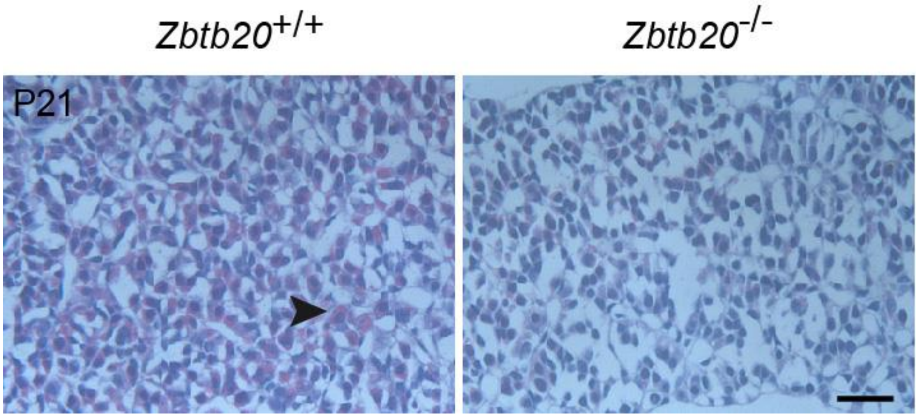

Supplementary Figure 2. The number of acidophilic cells decreases in *Zbtb20*$^{-/-}$ pituitary. Hematoxylin and eosin staining is performed in P21 *Zbtb20*$^{+/+}$ and *Zbtb20*$^{-/-}$ pituitaries. Arrowhead shows a typical acidophilic cell in anterior lobe. Scale bars: 30 μm.

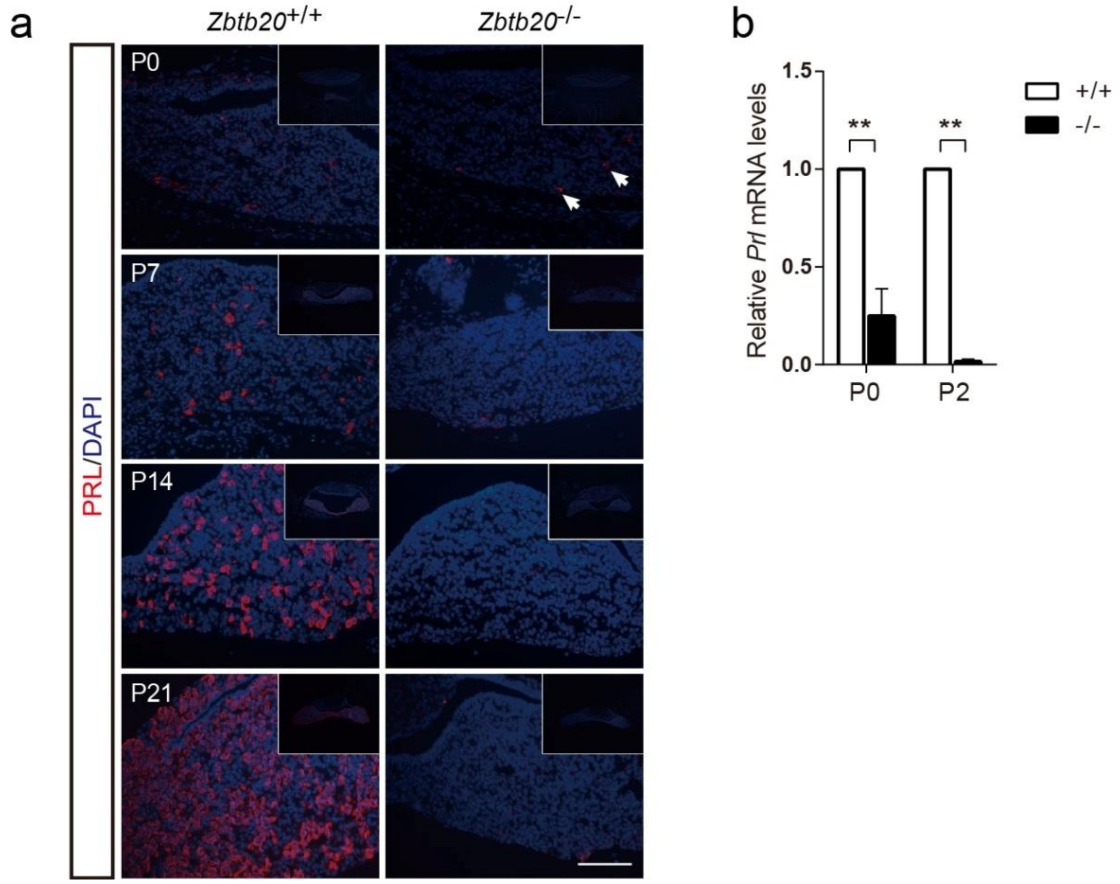

Supplementary Figure 3. Deficiency of lactotrope lineage in postnatal *Zbtb20*$^{-/-}$ pituitaries. (**a**) PRL expression in anterior pituitaries is detected by immunohistochemistry assay on 0- to 3-week-old *Zbtb20*$^{+/+}$ and *Zbtb20*$^{-/-}$ littermates, and the disappearance of PRL-positive cells in null mice occurs at the days after P0. Inserts show the whole coronal section of pituitaries. Arrows indicate PRL-positive cells. Scale bars: 100 μm. (**b**) Quantification of *Prl* mRNA level in P0 and P2 *Zbtb20*$^{+/+}$ and *Zbtb20*$^{-/-}$ mice by realtime RT-PCR. Results are presented as fold induction relative to mRNA levels in wildtype mice. A sample was pooled from 4 adenohypophyses of the same phenotype, and the experiment was repeated two times. Values represent mean ± s.e.m. ** $P < 0.01$ vs. +/+ (Student's *t*-test).

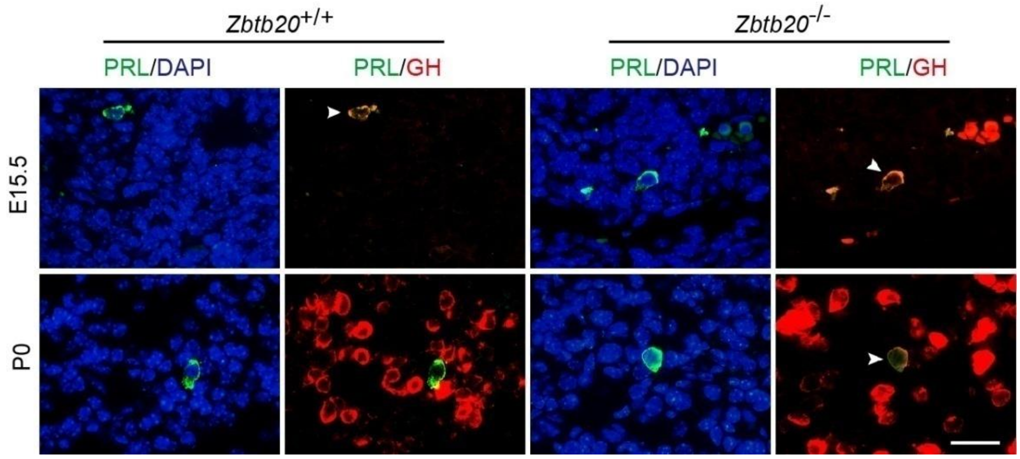

Supplementary Figure 4. PRL-positive cells including somatolactotropes are transiently present in Zbtb20-null pituitary in the perinatal period. Immunohistochemical double staining of PRL (green) and GH (red) in the pituitaries from *Zbtb20*$^{+/+}$ and *Zbtb20*$^{-/-}$ mice at the age between E15.5 to P0. Cell nuclei were counterstained with DAPI (blue). Arrowheads indicate PRL and GH double-positive cells. Scale bar: 25 μm

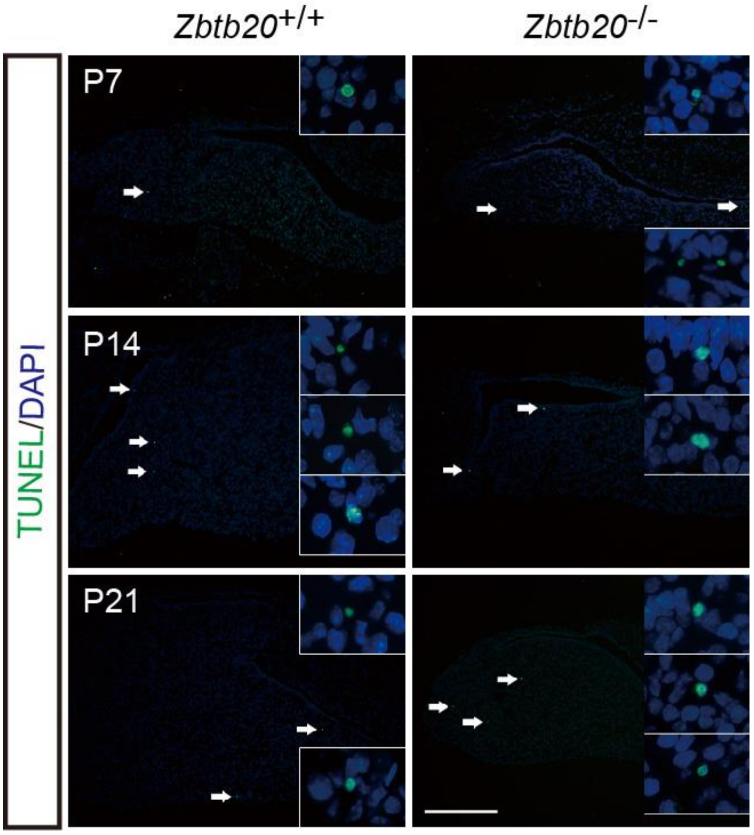

Supplementary Figure 5. ZBTB20 disruption does not affect apoptosis in anterior lobe cells. TUNEL is performed to label apoptotic cells in anterior lobe from *Zbtb20*$^{+/+}$ and *Zbtb20*$^{-/-}$ littermates at the indicated stages. Inserts are amplified apoptotic cells indicated by arrows. Scale bar: 200 μm.

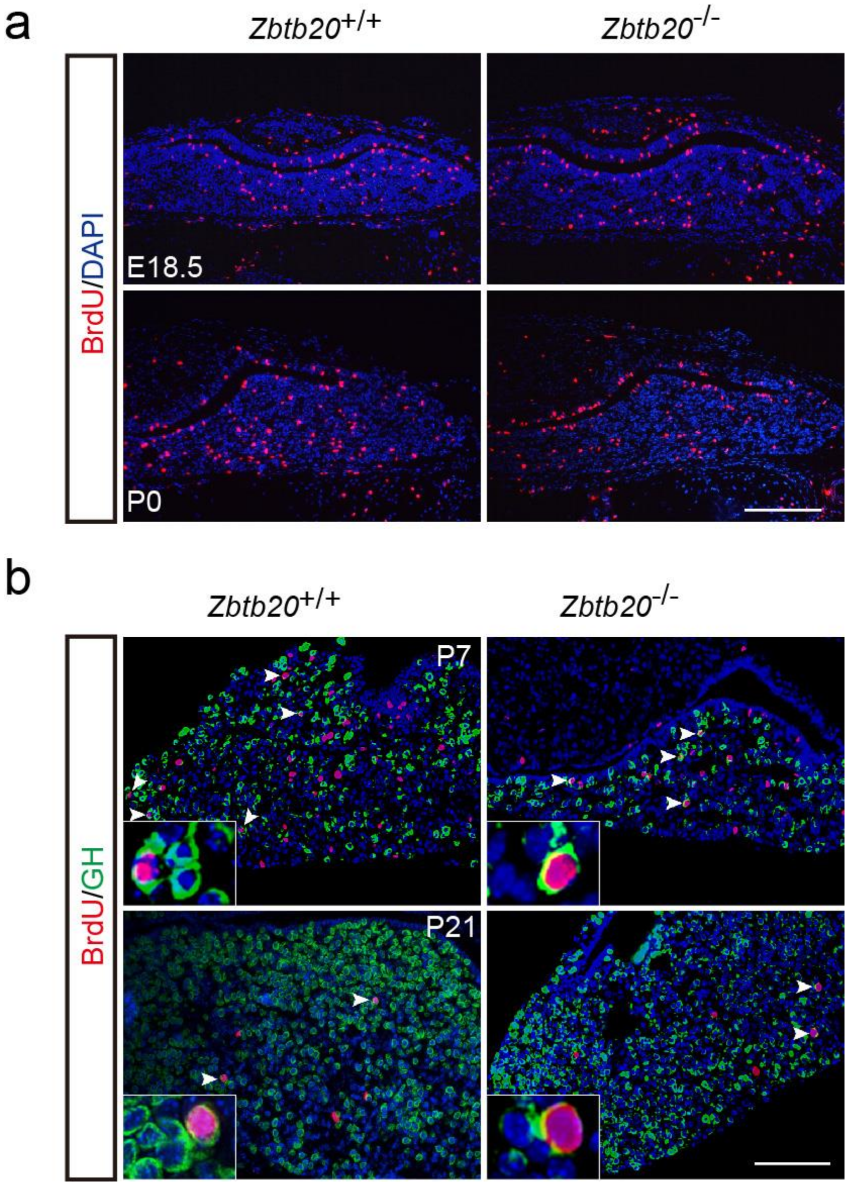

Supplementary Figure 6. Pituitary cell proliferation in in *Zbtb20*$^{-/-}$ mice. (**a**) Immunohistochemical staining of BrdU incorporation (red) was performed on the pituitaries of *Zbtb20*$^{+/+}$ and *Zbtb20*$^{-/-}$ littermates to assess cell proliferation at E18.5 and P0. ZBTB20 deletion does not affect pituitary cell proliferation in this perinatal period**.** (**b**) Immunohistochemical double staining of BrdU (red) and GH (green) was performed at the indicated ages of P7 and P21 in *Zbtb20*$^{+/+}$ and *Zbtb20*$^{-/-}$ pituitaries. Arrowheads indicate double-positive cells and inserts show the enlargement of double-positive cells. ZBTB20 deletion leads to a reduction in the proliferation of somatotropes at the ages. Scale bars: 200 μm for **a,** 100 μm for **b**.

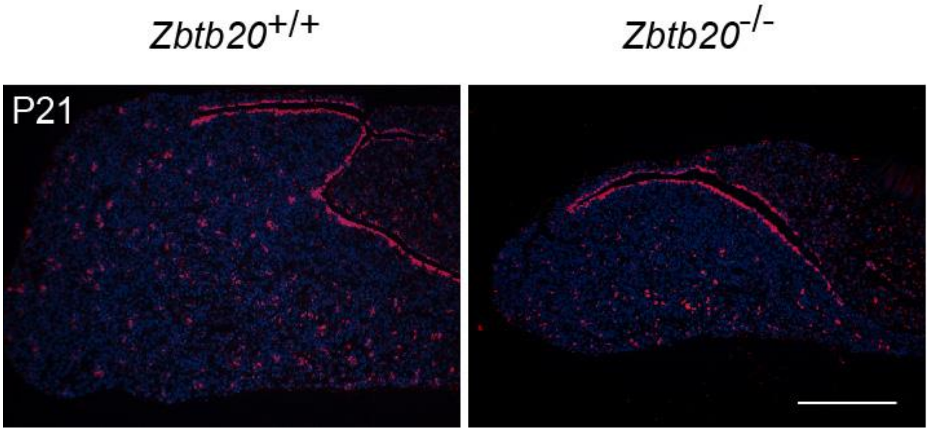

Supplementary Figure 7. ZBTB20 disruption does not affect the number and distribution of postnatal stem cells in anterior lobe. Immunohistochemical staining of Sox2 was performed on *Zbtb20*$^{+/+}$ and *Zbtb20*$^{-/-}$ pituitaries at P21. There is no significant difference in the number and distribution of Sox2-positive cells between the two genotypes. Scale bars: 200 μm.

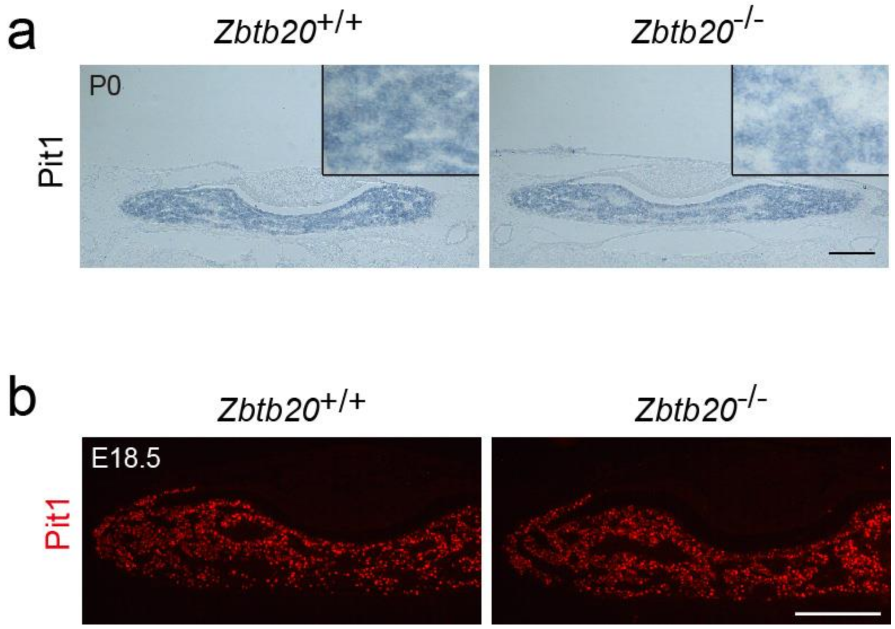

Supplementary Figure 8. Expression of Pit-1 is not altered by the loss of ZBTB20 in anterior pituitary at the perinatal stage. (**a**) In situ hybridization was performed using Pit-1 RNA probe on *Zbtb20*$^{+/+}$ and *Zbtb20*$^{-/-}$ pituitaries at P0. Inserts show higher magnification of the anterior lobe. (**b**) Immunohistochemical staining of Pit-1 was performed on *Zbtb20*$^{+/+}$ and *Zbtb20*$^{-/-}$ pituitaries at E18.5. Scale bars: 200 μm for **a** and **b**.

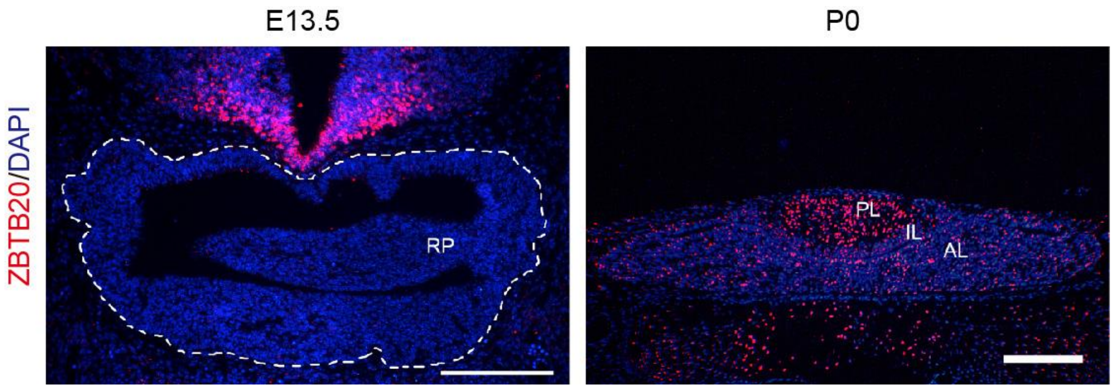

Supplementary Figure 9. Expression of ZBTB20 in the developing hypothalamus and pituitary. ZBTB20 expression was detected by immunohistochemical staining on normal mouse adenohypophysis at the indicated ages. White outline indicates Rathke's pouch. RP, Rathke's pouch; AL, anterior lobe; IL, intermediate lobe; PL, posterior lobe. Scale bars: 200 μm.

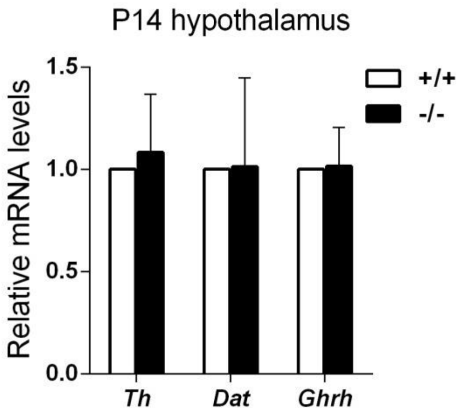

Supplementary Figure 10. ZBTB20 deletion does not alter mRNA expression levels of *Th*, *Dat*, or *Ghrh* in the hypothalamus. Quantification of *Th, Dat* and *Ghrh* mRNA levels in hypothalamus of 14-day-old *Zbtb20*$^{+/+}$ and *Zbtb20*$^{-/-}$ mice by realtime RT-PCR. Results are presented as fold induction relative to mRNA levels in control mice. Values represent mean ± s.e.m. n = 4. Student's *t*-test was performed for statistical analysis..

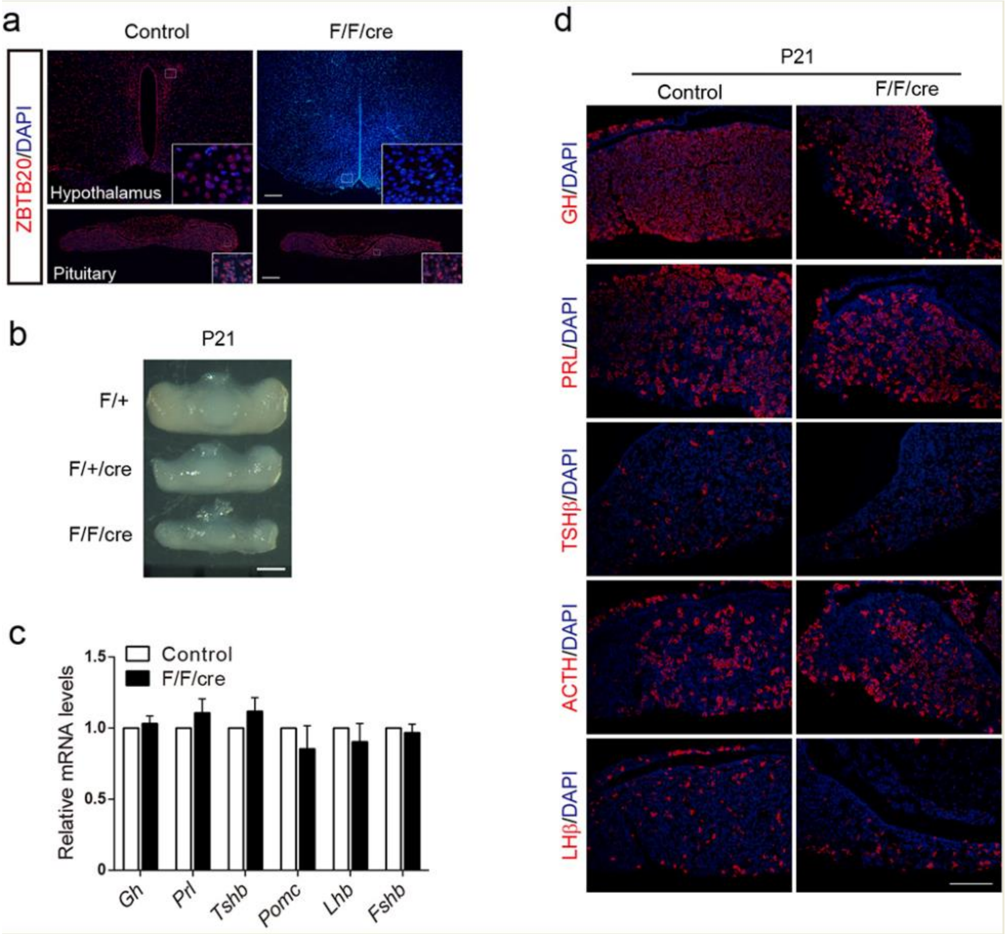

Supplementary Figure 11. Lactotrope specification is not affected in the mice lacking Zbtb20 specifically in nervous system. (**a**) ZBTB20 expression detected by immunohistochemical staining in pituitary and hypothalamus from 2-month-old mouse. Inserts are enlargement of the frame-indicated areas. Scale bars: 200 μm. (**b**) A general view of 21-day-old pituitaries from control, heterozygote and NS-ZB20KO littermate mice. Scale bar: 500 μm. (**c**) Quantification of hormone mRNA levels in 21-day-old NS-ZB20KO mice and control by realtime RT-PCR. Results are presented as fold induction relative to mRNA levels in control mice. A sample was pooled from 3-5 adenohypophyses of the same phenotype, and the experiment was repeated three times. (**d**) All five types of cell lineages in anterior pituitary are detected by immunohistochemical staining using GH, PRL, TSHβ, ACTH and LHβ antibodies in 21-day-old NS-ZB20KO mice and control littermates. Scale bar: 100 μm. Values represent mean ± s.e.m (Student's *t*-test).

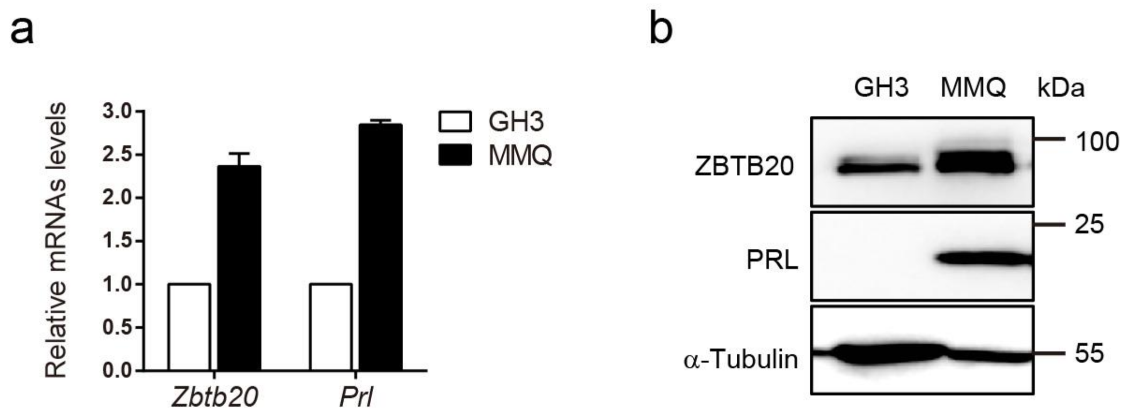

Supplementary Figure 12. Expressions of ZBTB20 and PRL in GH3 and MMQ cells. (**a**) Real-time RT-PCR was performed to detect *Zbtb20* and *Prl* mRNA levels. Results are normalized to *Gapdh* expression and presented as a fold relative to mRNA level in GH3 cells. $n = 3$. Values represent mean ± s.e.m. (**b**) Western blot was performed to detect ZBTB20 and PRL protein levels. After longer exposure, PRL band was visible in GH3 cell lysates. $n = 3$.

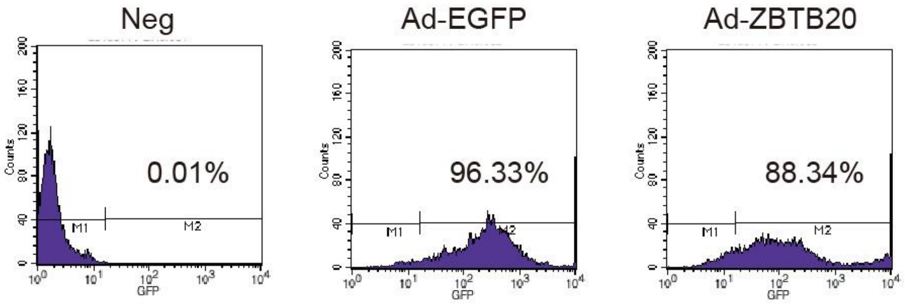

Supplementary Figure 13. Infection efficiency of GH3 cells with ZBTB20-expressing or mock adenovirus. GH3 cells were infected by recombinant replication-deficient adenoviruses co-expressing ZBTB20 and EGFP or mock adenoviruses expressing EGFP. Three days after infection, flow cytometry assay was performed to measure the percentage of EGFP-positive cells.

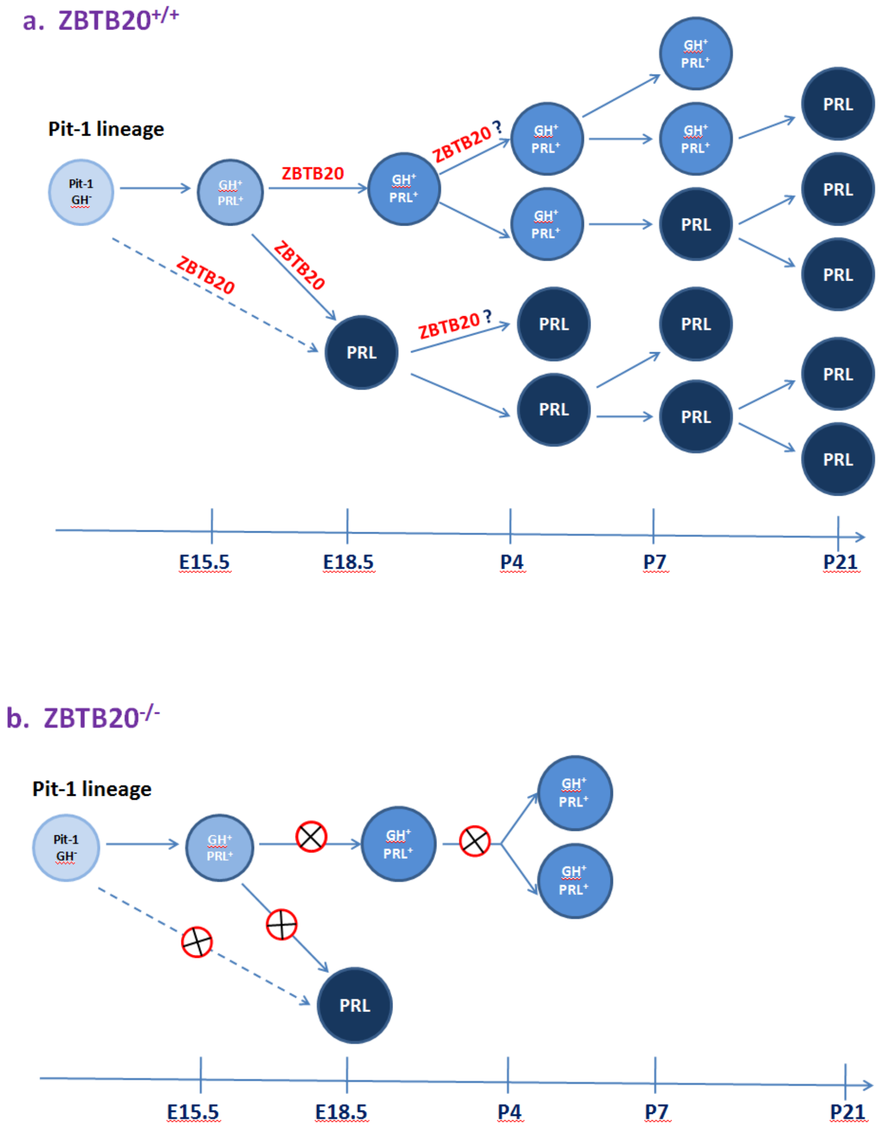

Supplementary Figure 14. Schematic demonstration of the regulation of lactotrope development by ZBTB20. (a) Under normal condition, somatolactotropes are generated from Pit-1 lineage in a ZBTB20-independent manner, and undergo cell differentiation and proliferation before being specified into lactotropes in a ZBTB20-dependent manner. Alternatively, lactotropes can be specified from somatolactotropes before the latter undergo cell replication, or even directly derived from GH-negative Pit-1 lineage, for both of which ZBTB20 is indispensable. (**b**) Disruption of ZBTB20 leads to complete blockage of lactotrope specification and lineage expansion. The developmental stages are indicated.

Figure 2d

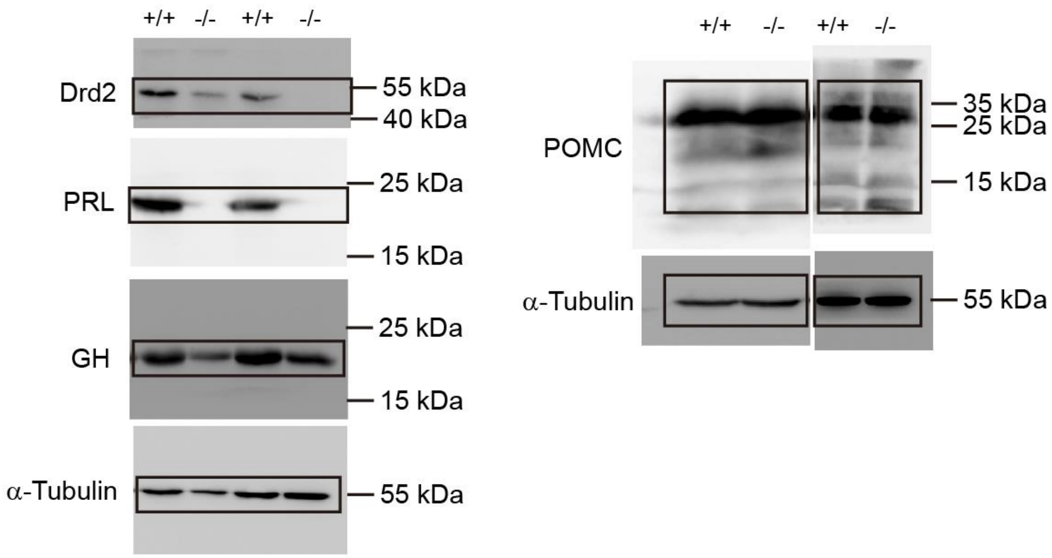

Supplementary Figure 15. Uncropped scans of Figure 2d.

Supplementary Table 1. Sequence of the primers used for quantitative RT-PCR analysis.

Gene	Orientation	Sequence (5' to 3')	PCR product
mIgf-1	Forward	CTGGACCAGAGACCCTTTGC	269 bp
	Reverse	GGACGGGGACTTCTGAGTCTT	
mPrl	Forward	CTCACTACATCCATACCCTG	242 bp
	Reverse	TTGAATACCACCTACTCCA	
rPrl	Forward	TCCCGGAGCTGTTTGACC	334 bp
	Reverse	AGCCGCTTGTTTTGTTCCTC	
Gh	Forward	CTTCTCGCTGCTGCTCATC	213 bp
	Reverse	TTGGCGTCAAACTTGTCATA	
mTshβ	Forward	GTAGTGGGTGGAGAAGAGT	180 bp
	Reverse	CAGATGGTGGTGTTGATG	
mPomc	Forward	AGCAACCCGCCCAAGG	61 bp
	Reverse	GCGTCTGGCTCTTCTCGG	
mLhβ	Forward	TGGCCGCAGAGAATGAGTT	166 bp
	Reverse	CCAGGGAGGCGGACAGA	
mFshβ	Forward	TGCCCGCCACTCAGACTCC	167 bp
	Reverse	GTGGGCGAACGGCAATGT	
Zbtb20	Forward	GCAGCCGGCAGCCCCTTCTTC	410 bp
	Reverse	CGCTCGCCGCTGCCATTCTG	
Pit-1	Forward	TCCCCAGAAATCCGAGAACT	350 bp
	Reverse	TGCGAGGAAGGCTTGCTGTGC	
mDrd2	Forward	ACCTGTCCTGGTACGATGATG	105 bp
	Reverse	GCATGGCATAGTAGTTGTAGTGG	
Erα	Forward	GCGGCATACGGAAAGACC	430 bp
	Reverse	ATGGAGCGCCAGACGAGAC	
Th	Forward	TTGAAGCCAAAATCCACC	141 bp
	Reverse	CAGACACCCGACGCACAG	
Dat	Forward	AAATGCTCCGTGGGACCAATG	173 bp
	Reverse	GTCTCCCGCTCTTGAACCTC	
Gapdh	Forward	AGGTCGGTGTGAACGGATTTG	123 bp
	Reverse	TGTAGACCATGTAGTTGAGGTCA	
